# Direct generation of time-energy-entangled W triphotons in atomic vapor

**DOI:** 10.1126/sciadv.ado3199

**Published:** 2024-09-13

**Authors:** Kangkang Li, Jianming Wen, Yin Cai, Saeid Vashahri Ghamsari, Changbiao Li, Feng Li, Zhaoyang Zhang, Yanpeng Zhang, Min Xiao

**Affiliations:** ^1^ Key Laboratory for Physical Electronics and Devices of the Ministry of Education & Shaanxi Key Lab of Information Photonic Technique, Xi'an Jiaotong University, Xi'an 710049, China.; ^2^ Department of Electrical and Computer Engineering, Binghamton University, Binghamton, NY 13902, USA.; ^3^ Department of Physics, Kennesaw State University, Marietta, GA 30060, USA.; ^4^ National Laboratory of Solid State Microstructures, College of Engineering and Applied Sciences and School of Physics, Nanjing University, Nanjing 210093, China.; ^5^ Department of Physics, University of Arkansas, Fayetteville, AR 72701, USA.

## Abstract

Entangled multiphoton sources are essential for both fundamental tests of quantum foundations and building blocks of contemporary optical quantum technologies. While efforts over the past three decades have focused on creating multiphoton entanglement through multiplexing existing biphoton sources with linear optics and postselections, our work presents a groundbreaking approach. We observe genuine continuous-mode time-energy-entangled W-class triphotons with an unprecedented production rate directly generated through spontaneous six-wave mixing (SSWM) in a four-level triple-Λ atomic vapor cell. Using electromagnetically induced transparency and coherence control, our SSWM scheme allows versatile narrowband triphoton generation with advantageous properties, including long temporal coherence and controllable waveforms. This advancement is ideal for applications like long-distance quantum communications and information processing, bridging single photons and neutral atoms. Most importantly, our work establishes a reliable and efficient genuine triphoton source, facilitating accessible research on multiphoton entanglement.

## Teaser

Reliable genuine W triphotons in continuous modes are directly generated with record efficiency in hot atoms via SSWM.

## INTRODUCTION

Generating entangled multiphoton states (*1*) is pivotal to probe quantum foundations and advance technological innovations. Comprehensive studies have already shown that multiphoton entanglement (*1*) enables a plethora of classically impossible phenomena, most of them incomprehensible with any bipartite system. Unfortunately, we hitherto have at hand only biphoton sources based on spontaneous parametric down-conversion (SPDC) or spontaneous four-wave mixing (SFWM). This has urged tremendous efforts on developing multiphoton sources (*1*–*3*) over the past 30 years. Among them, the most popular means is to multiplex existing biphoton sources with linear optics and postselections. This brings us the well-known exemplar of polarization-entangled multiphotons (*4*–*8*) by constructing imperative interferometric setups. Although postselection might be acceptable in some protocols, it is generally deleterious for most applications since the action of observing photons alters and destroys the states. To avoid postselection, the second path considers cascaded SPDCs/SFWMs (*9*–*12*) or two SPDCs/SFWMs followed by one up-conversion (*13*, *14*). In this way, polarization or time-energy entangled triphotons were reported by building sophisticated coincidence counting circuits. Despite no needs on interferometric settings, the attained states are intrinsically non-Gaussian due to unbalanced photon numbers between the primary and secondary biphoton process, thereby making these sources very noisy and inefficient. Alternatively, the third technique (*15*–*17*) suggests to coherently mix paired photons with singles attenuated from a continuous-wave (cw) laser to trigger triphoton events. Akin to the first method, this solution depends on erasing the photon distinguishability by resorting to the Hong-Ou-Mandal interference effect (*18*). Although polarization-entangled multiphotons of inequivalent classes were experimented with postselection, the low success rate and required interferometric stabilization make this proposal not so practical. As photons are always emitted in pairs in SPDC/SFWM, this attribute results in the fourth route (*19*–*23*) to make use of emission of multiple pairs by appropriately setting input pump powers. Although it seems easy to yield even-number states, yet, dominant biphotons from lower-order perturbation of the parametric process challenge detecting entangled multiphotons from higher-order perturbations. To have an acceptable fidelity, like the second way, a complicated detection system plus an interferometric setup is often inevitable in practice. What is more, this approach mainly allows to form polarization entanglement thus far. Despite these impressive achievements, all foregoing mechanisms are difficult to offer a reliable and efficient triphoton source for research and applications. In addition, so far there is no convincing realization of the entangled triphoton experiment in continuous modes. Driven by SPDC, one would expect that such photons could be naturally born from third-order SPDC (*24*–*26*) by converting one pump photon of higher energy into three daughter photons of low energy. The idea looks simple and straightforward, but experimentally inaccessible owing to the lack of such a nonlinear optical material. As a result, developing a reliable triphoton source is still in its infancy even up to today.

Coherent atomic media (*27*), on the other hand, exhibit a wide range of peculiar properties including giant nonlinearities, prolonged atomic coherence, strong photon-atom interaction, and slow/fast light effects. Recently, these exotic properties have been skillfully used to construct a novel narrowband biphoton source (*28*–*31*) basing on SFWM. Specifically, giant nonlinearities promise efficient parametric conversion, long atomic coherence leads to narrowband wave packets, and sharp optical response becomes a formidable knob for shaping photon waveforms and temporal correlations. Unlike solid-state sources, one unique feature pertinent to atomic ensembles arises from the dual role played by the third-order nonlinear susceptibility χ^(3)^ in biphoton generation (*28*, *32*–*34*). That is, in addition to governing nonlinear conversion strength, the double-resonance structure in χ^(3)^ signifies the coexistence of two sets of SFWMs in light quanta radiation. Alternatively, entangled photons output from these two stochastic but coherent SFWM processes interfere and give rise to a nontrivial two-photon interference, namely, the damped Rabi oscillations. In general, their waveforms are entirely patterned by the convolution of a complex phase-mismatch function and χ^(3)^. Other than these attributes, the nonclassical correlations shared by paired photons can be additionally manipulated by exploiting various coherent control techniques including electromagnetically induced transparency (EIT) (*27*) to reshape optical responses. The interplay among diverse effects also enriches fundamental research and fosters technological innovations, inaccessible to other existing biphoton sources. Besides, flexible system layouts like backward detection geometry are more favorable to photon counting detection. Motivated by these advantages, here, we move one step forward and report the direct generation of continuous-mode triphotons entangled in time and energy from a hot atomic vapor cell. By using the process of spontaneous six-wave mixing (SSWM) (*35*, *36*), we have not only observed the notable three-photon interference but also witnessed the residual two-photon correlation by tracing one photon out, an intrinsic virtue of the W class of tripartite entanglement (*37*). By adjusting the system parameters, we have further achieved waveform-controllable triphoton generation. Together with an unprecedented production rate, our scheme has substantiated to be the first reliable platform that leverages multipartite entanglement research to an unparalleled level.

## RESULTS

**Fig. 1.**
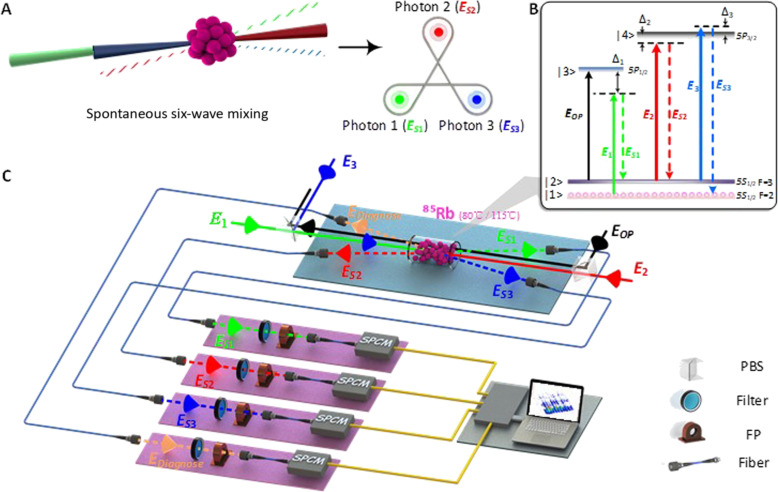
Generation of genuine W triphotons entangled in time-energy directly via SSWM in a hot atomic vapor. (**A**) Conceptual schematic of creating a W triphoton state via the fifth-order parametric nonlinear process. (**B**) The ^85^Rb energy-level diagram of the SSWM process. (**C**) The experimental setup. Three coaxial input driving fields *E*_1_ (795 nm), *E*_2_ (780 nm), and *E*_3_ (780 nm) are coupled into the center of an ^85^Rb vapor cell heated at 80°C (or 115°C) to initiate the simultaneous generation of W triphotons in *E*_*S*1_, *E*_*S*2_, and *E*_*S*3_. An additional optical-pumping beam *E*_OP_ is added to clean up the residual atomic population in the level |2 > for preventing the noise from the Raman scattering. The generated photons are coupled into a data acquisition system by single-mode fibers and jointly detected by three synchronized single-photon counting modules (SPCMs) with filters (F) and Fabry-Perot cavities (FP) placed in front. To eliminate accidental coincidences caused by dual biphotons and quadraphotons, an extra detection of the diagnosis photons *E*_Diagnose_ is applied to ensure the natural triphoton collection. All trigger events are then interrogated by a fast-time acquisition card with a computer.

As schematic in Fig. 1 (A to C), we are interested in yielding narrowband W triphotons from a 7-cm-long ^85^Rb vapor cell with a four-level triple-Λ atomic configuration at temperature 80°C (or 115°C). The detail of the experimental setup is provided in Methods. In the presence of three counter-propagating cw laser beams [one weak pump ( $E_{1},\omega_{1},{\overset{⃑}{k}}_{1}$ ) and two strong couplings ( $E_{2},\omega_{2},{\overset{⃑}{k}}_{2}$ ) and ( $E_{3},\omega_{3},{\overset{⃑}{k}}_{3}$)], backward photon triplets ( $E_{\mathrm{Sj}},\omega_{\mathrm{Sj}},{\overset{⃑}{k}}_{\mathrm{Sj}}$ with *j* = 1,2,3) are emitted via Doppler-broadened SSWM at an intersection angle of θ ≈ 4° to the principle *z* axis along the phase matching direction, $\text{∆}\overset{⃑}{k}=\left({\overset{⃑}{k}}_{S1}+{\overset{⃑}{k}}_{S2}+{\overset{⃑}{k}}_{S3}\right)-\left({\overset{⃑}{k}}_{1}+{\overset{⃑}{k}}_{2}+{\overset{⃑}{k}}_{3}\right)=0$ . As depicted in Fig. 1 (B and C), the three coaxial input lasers were coupled into the center of the ^85^Rb vapor cell with tunable frequency detunings ∆*_j_* and powers *P_j_*; while the generated photon triplets were accordingly detected by three single-photon counting modules (SPCM_1_ to SPCM_3_) for coincidence counts after spatial and frequency filtering. Here, to avoid unwanted accidental trigger events induced by singles and dual biphotons, we placed single-band filters and narrowband etalon Fabry-Perot cavities in front of SPCM*_j_* before detection. We notice that in three-photon joint clicks, the major source of accidental coincidences stems from double pairs from two different SFWMs simultaneously present in the detection system (Supplementary Materials). Since these dual pairs may have similar central frequencies and polarizations as genuine triphoton modes, they cannot be filtered away simply by polarizers and frequency filters. To exclude such double-pair false trigger events, in experiment, we further introduced an additional SPCM_d_ synchronized with SPCM_3_ to serve as the diagnosis detector in conjunction with the rest two, SPCM_1_ and SPCM_2_. To ensure the atomic population to be mainly distributed in the ground level $|5S_{\frac{1}{2}},F=2\rangle$ throughout the measurement, an additional strong optical repumping beam (*E*_op_) was applied to the atomic transition $\left.|5S_{\frac{1}{2}},F=3\right\rangle \to \left.|5P_{\frac{1}{2}}\right\rangle$ in alignment with *E*_2_ but without spatial overlap. With these preparations, we carefully adjust the system parameters, especially *P_j_* and ∆*_j_* of each input field *E_j_*, to promote the SSWM occurrence.

Physically, the SSWM process can be understood from the effective interaction Hamiltonian

$$\begin{array}{c} H=\epsilon_{0}\int_{V}d^{3}r\chi^{\left(5\right)}E_{1}E_{2}E_{3}E_{S1}^{\left(-\right)}E_{S2}^{\left(-\right)}E_{S3}^{\left(-\right)}+\\ H.c.\left(H.c.,\text{Hermitian conjugate}\right) \end{array}$$ (1)

with three input (output) beams treated as classical (quantized) fields and *V* being the interaction volume. In Eq. 1, χ^(5)^ denotes the fifth-order Doppler-broadened nonlinear susceptibility and governs the nonlinear conversion efficiency. In the Schrödinger picture, after some algebra, the triphoton state at the two cell surfaces can be derived from first-order perturbation theory by ignoring the vacuum contribution (Supplementary Materials) and takes the form of

$$|\Psi \rangle \propto ∭d\omega_{S1}d\omega_{S2}d\omega_{S3}\chi^{\left(5\right)}\Phi \left(\frac{∆\mathrm{kL}}{2}\right)\delta \left(∆\omega \right)|1_{\omega_{S1}},1_{\omega_{S2}},1_{\omega_{S3}}\rangle$$ (2)

Here, $∆\omega =\sum_{j=1}^{3}\left(\omega_{\mathrm{Sj}}-\omega_{j}\right)$ , *L* is the interaction length, $\text{∆}k=\text{∆}\overset{⃑}{k}\cdot \hat{z}$ is the phase (or wave number) mismatch, the phase-mismatch longitudinal function Φ(*x*) = sinc (*x*)*e*^−*ix*^ ascribes the three-photon natural spectral width arising from their different group velocities. Besides conditioning the triphoton output rate, the χ^(5)^-resonance profile also specifies the generation mechanism along with the photon intrinsic bandwidths. Overall, the state (*2*) outlines a few peculiar features yet to be experimentally verified: First, because of its nonfactorization, ∣Ψ⟩ is entangled in frequency (or time), instead of polarization. Second, characterized by two independent variables, ∣Ψ⟩ conforms to the essential characteristics of the tripartite W class, that is, by tracing one photon away, partial entanglement still exists in the remaining bipartite subsystem. Third, since the triphoton waveform is defined by the convolution of Φ and χ^(5)^, two distinct types of Glauber third-order (as well as conditional second-order) temporal correlations are expected to be manifested in threefold (and conditioned twofold) coincidence counting measurement. Consequently, two very differing scenarios are expected to be revealed in triphoton coincidence counting measurement. Last, but not the least, the triplet production rate is linear in the intensity of each input laser and can be markedly enhanced by orders of magnitude by optimizing system parameters. It is worth pointing out that all these notable properties have been well affirmed in our series of experiments. Of importance, this is the first experimental proof of the time-energy-entangled triphoton W state found a decade ago (*37*) but never realized.

### Experimental set up

In experiment, we optimized the SSWM phase-matching condition via controlling the frequency detunings and incident angles of three driving fields so as to effectively collect emitted triphotons. Upon triggering SPCM*_j_*, the temporal correlation was concealed in photon-counting histograms saved in a fast-time acquisition card with 0.0244-ns bin width, where, within in every time window of 195 ns, the detection of an *E*_*S*1_-photon triggered the start of a coincidence event that ended with the detection of subsequent *E*_*S*2_- and *E*_*S*3_-photons. In most measurements, we collected the total trigger events over an hour and then analyzed the corresponding three-photon coincidences from the histogram in the parameter space (τ_21_, τ_31_), where τ_21_ = τ_2_ − τ_1_ and τ_31_ = τ_3_ − τ_1_ are respectively the relative time delays with τ*_j_* being the triggering time of the SPCM*_j_*.

### Three-dimensional triphoton temporal correlation

**Fig. 2.**
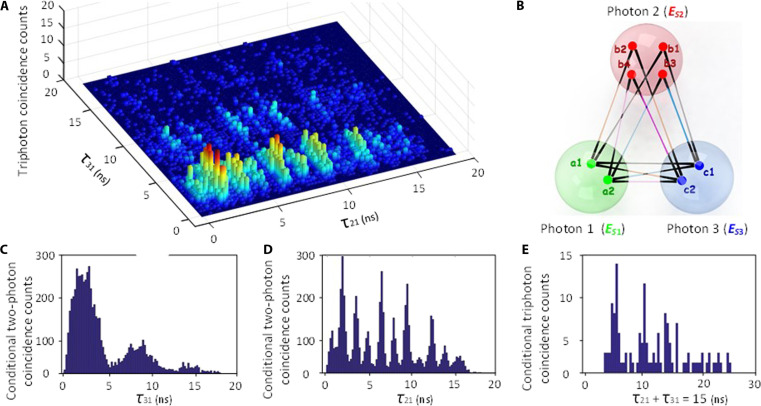
Triphoton coincidence counting measurements. (**A**) Three-dimensional (3D) quantum interference formed by three-photon coincidence counts collected in 1 hour with the time-bin width of 0.25 ns for OD = 4.6. The generation rate and accidentals are 102 ± 9 and 6 ± 1 per minute, respectively. The powers of the input *E*_1_, *E*_2_, and *E*_3_ beams are *P*_1_ = 4 mW, *P*_2_ = 40 mW, and *P*_3_ = 15 mW, respectively, and the corresponding frequency detunings are ∆_1_ = −2 GHz, ∆_2_ = −150 MHz, and ∆_3_ = 50 MHz. (**B**) Schematic illustration of triphoton interference originating from the coexistence of multi-SSWMs. (**C** and **D**) Conditional two-photon coincidence counts as the function of τ_21_ and τ_31_ in (A) by tracing the third photon *E*_*S*3_ and *E*_*S*2_, respectively. (**E**) Conditional three-photon coincidence counts along the trajectory of τ_21_ + τ_31_ = 15 ns in (A), as an illustration of flexible triphoton temporal correlation reading through slicing.

As an exemplar of such, Fig. 2A displays one set of measured threefold coincidence counts from one recorded histogram after subtracting the accidental noise, giving rise to an intriguing three-dimensional (3D) temporal correlation with the 18.6- and 19.0-ns effective measurement time window along the τ_21_ and τ_31_ axes because of the used detectors. For the 0.25-ns time bin width per detector, integrating all involved time bins yields the total of ~6 × 10^3^ threefold trigger events, which result in a raw triphoton generation rate of 102 ± 9 per minute without account of the coupling loss and detection efficiency. This rate is orders of magnitude higher than any previous one and can be further improved by applying more efficient SPCMs and optimizing the fiber coupling efficiency. From the raw data, the background accidentals were estimated to be 6 ± 1 per minute, mainly originating from the residual dual pairs and accidental coincidences of uncorrelated singles and dark counts of the SPCMs. This low background noise implies that the undesired third-order nonlinear processes were well filtered out in the experiment. On the other hand, the complicated 3D pattern is a direct consequence of nontrivial W triphoton interferences due to the occurrence of multiple coexisting SSWM processes in the regime of damped Rabi oscillations. As described previously, these processes arise from the multiresonance structure of χ^(5)^. According to our qualitative dressed-state calculations (Supplementary Materials), there are four such coexisting channels, as schematic in Fig. 2B, coherently contributing to the observed quantum interference. To confirm that the emitted triphoton state belongs to the W class, we then used the acquired data to investigate the correlation properties of different bipartite subsystems. To do so, we integrated the coincidence counts by tracing away one photon from every triphoton event over that photon’s arrival time. In this way, we acquired the conditional two-photon temporal waveforms with τ_21_ or τ_31_ as variables, and plotted them, respectively, in Fig. 2 (C and D). The conditioned τ_3_-waveform in Fig. 2D exhibits a damped periodic oscillation with a period of ~6.2 ns (Supplementary Materials), while the τ_21_-waveform in Fig. 2C reveals two superimposed damped periodic oscillations with another 1.7-ns period in addition to the 6.2-ns one (Supplementary Materials), an interference effect unusual to any existing biphoton source. In contrast, the 3D triphoton waveform has flexible temporal widths and is subject to the slicing, for instance, 28 ns along the direction of τ_21_ + τ_31_ = 15 ns (Fig. 2E). This contrasting phenomenon also supports our theoretical picture from alternative aspect that the observed interference is caused by at least three sets of coherently coexisting SSWM processes. As demonstrated in the Supplementary Materials, our qualitative analysis gives a good account of the experimental data.

**Fig. 3.**
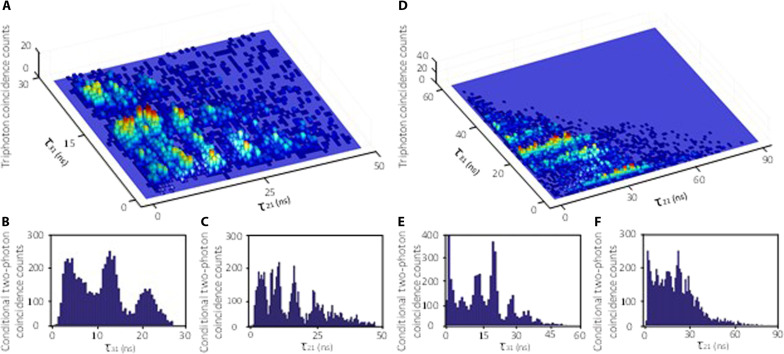
Triphoton coincidence counting measurements by tuning the coupling strength and OD. (**A**) 3D quantum interference formed by three-photon coincidence counts collected in 1 hour with the 0.7-ns time-bin width by changing *P*_2_ to 15 mW and ∆_2_ to −50 MHz. Other parameters are same as Fig. 2. The generation rate and accidentals rate are 77.4 ± 7.8 and 11 ± 2.1 per minute, respectively. (**B** and **C**) Conditional two-photon coincidence counts as the function of τ_21_ and τ_31_ in (**A**) by tracing the third photon *E*_*S*3_ and *E*_*S*2_, respectively. (**D**) Collected over 40 min with 1-ns time-bin width by changing OD to 45.7. Other parameters are same as Fig. 2. The generation and accidentals rates are 125 ± 11 and 28 ± 6.4 per minute, respectively. (**E** and **F**) Conditional two-photon coincidence counts as the function of τ_21_ and τ_31_ in (**D**).

Since the attributes of triphoton waveforms are dependent on the system parameters, this prompts us to manipulate and control their quantum correlations by means of tuning the input lasers and the atomic density or optical depth (OD). To this end, we carried out a series of experiments to tailor temporal correlation by shaping their waveforms by varying various parameters. Two sets of such representative experimental data are presented in Fig. 3. In comparison to Fig. 2A, Fig. 3A shows the steered waveform by reducing the power and frequency detuning of the input *E*_2_ laser. As one can see, the profile of the triphoton temporal correlation is markedly changed despite the reduced generation rate 77.4 ± 7.8 min^−1^. Especially, the conditional two-photon coincidence counts manifest monoperiodic oscillations with the same period of 6.2 ns along both τ_21_ and τ_31_ directions, as illustrated in Fig. 3 (B and C). This is because, in this case, the Rabi frequency of *E*_2_ was tuned to be very close to that of *E*_3_. As a consequence, half of the multiple resonances associated with the emission of *E*_*S*2_-photons (Fig. 2B) become degenerate and share the same spectrum. Likewise, the triphoton temporal coherence length along the τ_21_ + τ_31_ = 29 ns direction is enlarged to 40 ns. On the other hand, triphoton interference can be also modulated by altering the phase-mismatch longitudinal function Φ in Eq. 2. Akin to the biphoton generation, the phase mismatch ∆*k* in Φ is determined by the linear susceptibility of each mode in SSWM via the EIT slow-light effect. As showcased in Fig. 3D, by augmenting the OD from 4.6 to 45.7, the triphoton temporal correlation is considerably modified by the dispersion relation of the atomic vapor and falls into the group-delay regime. In addition to raising the production rate to 125 ± 11 per minute, the oscillatory curvature is markedly suppressed and replaced by the overall decay envelopes. This transformation becomes more evident when examining the conditioned two-photon coincidence counts. By comparing Fig. 3F with Fig. 3 (B, C, and E), one can see that the enhanced dispersion apparently smears the damped Rabi oscillations along the τ_21_-direction, implying that the narrower bandwidths defined by $\Phi \left(\frac{\text{∆}\mathrm{kL}}{2}\right)$ regulate the bandwidths dictated by χ^(5)^ to obscure the interference amongst four sets of coexisting SSWM channels. Besides, the triphoton temporal coherence length along the direction of τ_21_ + τ_31_ = 50 ns is also significantly prolonged up to 70 ns.

**Fig. 4.**
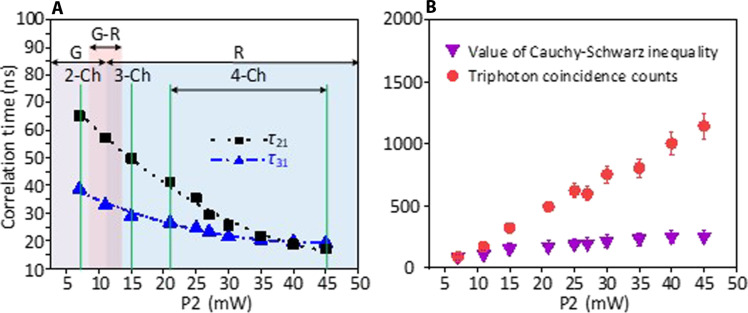
Controllable waveform generation and the violation of the Cauchy-Schwarz inequality. (**A**) The correlation times of conditional two-photon coincidences along the τ_21_ (black squares) and τ_31_ (blue triangles) directions by changing *P*_2_. By increasing *P*_2_, the triphoton temporal correlation is shifted from the group-delay (G) regime to the Rabi-oscillation (R) region. *j*-Ch (*j* = 2,3,4) means the coherent coexistence of *j* types of indistinguishable SSWMs. The experimental condition is same as that in Fig. 2. (**B**) The violation of the Cauchy-Schwarz inequality (purple inverted triangles) and the triphoton generation rates (red dots) in 15 min verses the input power *P*_2_ of the driving field *E*_2_*.*

To reveal the nonclassicality of the W triphoton state, we continued to examine the violation of the Cauchy-Schwarz inequality (*38*, *39*) as well as the fringe visibilities of the observed Rabi oscillations in Fig. 4 (A and B). By normalizing the threefold coincidence events to the flat background counts along with the additional autocorrelation measurement of the collected *E*_*S*1_, *E*_*S*2_, and *E*_*S*3_ photons, we found that the Cauchy-Schwarz inequality is violated by a factor of 250 ± 55 in Fig. 2A, 154 ± 43 in Fig. 3A, and 79 ± 21 in Fig. 3D. Note that here, these values were optimized by filtering possible biphoton processes in measurement. As shown in Fig. 4B, we found that the violation of the Cauchy-Schwarz inequality increases gradually with the rise in power *P*_2_. In addition, we observed that the fringe visibility in Fig. 2A can be as high as 90 ± 5%.

In addition to the above experiments, it is also instructive to explore the triphoton production rate and temporal correlation width as a function of the input pump power for further understanding of the proposed generation mechanism. This has motivated us to implement additional measurements and the experimental data are presented in Fig. 4A. As one can see, indeed, the triphoton generation rate follows a linear growth in the input power *P*_2_ of the *E*_2_ field. For the temporal coherence length, we concentrated on the two-photon conditional coincidence counting along the τ_21_ and τ_31_ directions. From Fig. 4A, it is not difficult to find that increasing *P*_2_ results in the reduction of the correlation time. This stems from the reduced slow-light effect when augmenting *P*_2_. Note that Figs. 2A and 3 (A and D) simply become one individual point in Fig. 4. Overall, our approach enables all-optical coherent manipulation to create the genuine triphotons with controllable waveforms.

## DISCUSSION

In conclusion, we have observed the efficient dependable continuous-mode W triphoton emission directly through SSWM in a warm atomic vapor with a generation rate of about 125 ± 11 min^−1^. Moreover, because of the coexistence of multi-SSWMs, these time-energy-entangled W triphotons have resulted in various nontrivial three-photon temporal interferences. Furthermore, by manipulating the system parameters, the triphoton temporal correlations can be flexibly engineered and tailored and demonstrate many peculiar characteristics inaccessible to all previous mechanisms. As a reliable source, it is expected to play a vital role in probing foundations of quantum theory and advancing various quantum-based technologies in information processing, communications, networking, imaging, metrology, etc.

## METHODS

### Experimental implementation

Experimentally, three coaxial driving beams *E*_1_, *E*_2_, and *E*_3_ are coupled to the center of the ^85^Rb vapor cell to initiate the SSWM process, as shown in Fig. 2. The relevant energy-level diagram is shown in Fig. 1B, where the atoms are prepared at the ground level ∣1⟩ (5*S*_1/2_, *F* = 2). The other involved energy levels are ∣2⟩ (5*S*_1/2_, *F* = 3), ∣3⟩ (5*P*_1/2_), and ∣4⟩ (5*P*_3/2_). The horizontally polarized weak probe *E*_1_ beam at the 795-nm wavelength is applied the atomic transition ∣1⟩ → ∣3⟩ with a large red frequency detuning ∆_1_ (2 GHz) so that the atomic population resides primarily at ∣1⟩. The other two strong coupling beams *E*_2_ (780 nm, horizontal polarization) and *E*_3_ (780 nm, vertical polarization) are near resonantly coupled to the same atomic transition ∣2⟩ → ∣4⟩ but with changeable detunings ∆_2_ and ∆_3_. By carefully adjusting the phase matching conditions, the spatially separated triphotons *E*_*S*1_, *E*_*S*2_ and *E*_*S*3_ with wave vectors ${\overset{⃑}{k}}_{S1}$ , ${\overset{⃑}{k}}_{S2}$ , and ${\overset{⃑}{k}}_{S3}$ are spontaneously emitted along the phase-matching directions with a small forward angle about 4° away from the three driving fields. Besides, we have added an additional optical-pumping beam *E*_OP_ to clean up the residue atomic population in ∣2⟩ so that the Raman scattering can be suppressed from the transition ∣2⟩ → ∣3⟩. To increase the fifth-order nonlinearity, the ^85^Rb vapor cell with a length of *L* = 7 cm is heated to 80°C (or 115°C). In this regard, the reported data in Figs. 2 and 3 (A to C) were collected at the temperature of 80°C, while the data presented in Fig. 3 (D to F) were obtained at 115°C. Also, the narrowband filters and customized interference etalon Fabry-Perot cavities are placed in front of each SPCM to filter the scattered driving lasers from the collected triphoton trigger events. After being detected by SPCMs, the trigger events are recorded by a time-to-digit converter, where the maximum resolution time of our recording card is 813 fs. In our experiment, the fiber-fiber coupling efficiency and the SPCM detection efficiency are 70 and 40%, respectively.

### Filtering possible biphoton processes from triphoton coincidence counts

Although the triphoton generation by SSWM is the focus of the measurement, because of the larger magnitude of the third-order nonlinearity, it is necessary to consider the possible false counts from the biphoton processes. On the basis of the atomic-level structure and the adopted field coupling geometry, there are seven crucial SFWMs (fig. S6) that may result in accidental coincidences: (i) SFWM1 initiated by *E*_1_ and *E*_2_, (ii) SFWM2 by *E*_1_ and *E*_3_, (iii) SFWM3 by *E*_2_ and *E*_3_, (iv) SFWM4 by *E*_3_ and *E*_2_, (v) SFWM5 by 2*E*_1_, (vi) SFWM6 by 2*E*_2_, and (vii) SFWM7 by 2*E*_3_. Specifically, the biphotons produced from the following SFWMs may contribute to the accidental joint-detection probability: (i) SFWM1 + SFWM2, (ii) SFWM1 + SFWM3, (iii) SFWM1 + SFWM4, (iv) SFWM1 + SFWM5, (v) SFWM1 + SFWM7, (vi) SFWM2 + SFWM3, (vii) SFWM2 + SFWM4, (viii) SFWM2 + SFWM6, (ix) SFWM3 + SFWM4, (x), SFWM3 + SFWM5, (xi) SFWM3 + SFWM7, (xii) SFWM4 + SFWM5, (xiii) SFWM4 + SFWM7, (xiv) SFWM5 + SFWM6, and (xv) SFWM6 + SFWM7. Fortunately, the central frequency differences of the similar photons from SSWM and SFWMs are more than 3 GHz. Therefore, before being detected by SPCMs, the collected photons need to pass through the high-quality single-frequency band filters and the customized narrowband etalon Fabry-Perot cavity (with a bandwidth ~600 MHz). The bandwidth, transmission efficiency, and extinction ratio of the used filters are 650 MHz, 80%, and 60 dB, respectively. After these measures, most of the biphoton noise can be filtered from the detection. In addition, the phase-matching condition for the SSWM process is much different from those for the possible SFWM processes. For instance, the photons from SFWM2 have distinctive emission angles from those from SSWM. As a result, the three-photon coincidence counts in actual measurements are mainly determined by true triphotons, uncorrelated singles, and dark counts. In practice, the biphotons and uncorrelated singles can be well filtered in the three-photon coincidence counting measurement by carefully adjusting the phase-matching conditions.

### Additional detection of diagnosis photons *E*_Diagnose_

To further guarantee the detected photons that are really from SSWM, we have performed one additional detection of the two-photon coincidences *E*_*S*3_ and *E*_Diagnose_ simultaneously in conjunction with the coincidences between *E*_*S*1_ and *E*_*S*2_ by artificially introducing the diagnose photons *E*_Diagnose_. This arrangement allows us to greatly reduce the false three-photon trigger events from dual biphotons particularly. The experimental results of *E*_*S*3_ and *E*_Diagnose_ are given in the Supplementary Materials. By the same reconstruction method, we notice that the trigger events from two pairs of biphotons can be safely removed from the data recording.

It is worth pointing out that the diagnosis detector SPCM_d_ in our detection system is used to exclude “false three-photon events” from dual biphotons, uncorrelated singles, and dark counts of the SPCMs. However, it is not used for postselection as required in some indirect methods mentioned in the introduction of the main text. Instead, it actually serves as an additional diagnostic layer for excluding accidental coincidences in our experiments.

### The Cauchy-Schwarz inequality

The nonclassicality of triphoton correlation can be verified by observing the violation of the well-known Cauchy-Schwarz inequality, which is defined by

$$\frac{{\left[g^{\left(3\right)}\left(\tau_{21},\tau_{31}\right)\right]}^{2}}{\left[g_{S1}^{\left(2\right)}\right]\left[g_{S2}^{\left(2\right)}\right]\left[g_{S3}^{\left(2\right)}\right]}\leq 1$$

Here, *g*^(3)^(τ_2_, τ_3_) is the normalized third-order correlation function with respect to the accidental background. $g_{S1}^{\left(2\right)}$ , $g_{S2}^{\left(2\right)}$ and $g_{S3}^{\left(2\right)}$ are the normalized autocorrelations of the emitted photons *E*_*S*1_, *E*_*S*2_, and *E*_*S*3_ measured by a fiber beam splitter. In our experiment, the nonzero background floor such as in Figs. 2 and 3 is a result of the accidental coincidences between uncorrelated single photons. According to the measured data, we estimate that the maximum values of $g_{S1}^{\left(2\right)}$ , $g_{S2}^{\left(2\right)}$ , and $g_{S3}^{\left(2\right)}$ are respectively to be 1.6 ± 0.2, 2, and 2.

## Supplementary Materials

### This PDF file includes:

Supplementary Text S1 to S3 Figs. S1 to S13 Table S1 References
