# Supplementary material for: Direct generation of time-energy-entangled W triphotons in atomic vapor
Source: Sci Adv. 2024 Sep 13;10(37):eado3199. doi: 10.1126/sciadv.ado3199 (PMC11397414; doi:10.1126/sciadv.ado3199)
Supplement: Supplementary file 1 — Supplementary Text S1 to S3 Figs. S1 to S13 Table S1 References [file sciadv.ado3199_sm.pdf]

Science Advances
AAAS

Supplementary Materials for

Direct generation of time-energy-entangled W triphotons in atomic vapor

Kangkang Li *et al.*

Corresponding author: Jianming Wen, jianming.wen@gmail.com; Yin Cai, caiyin@xjtu.edu.cn;
Yanpeng Zhang, ypzhang@mail.xjtu.edu.cn.

Sci. Adv. **10**, eado3199 (2024)
DOI: 10.1126/sciadv.ado3199

This PDF file includes:

Supplementary Text S1 to S3
Figs. S1 to S13
Table S1
References

Supplementary Text

I. Qualitative Theory of Time-Energy-Entangled W Triphoton Generation in Atomic Vapor

Qualitative Derivation of Fifth-Order Nonlinear Susceptibility $\chi^{(5)}$

Nonlinear optics stands as a foundational pillar in the realm of generating, shaping, and transforming quantum light. In the pursuit of harnessing nonclassical light through the deployment of atomic ensembles, the optical response of these systems, encompassing both linear and nonlinear susceptibilities, emerges as a pivotal determinant shaping the characteristics of the resultant quantum states and waveforms. This influence is particularly pronounced when the interaction between light and atoms transpires in proximity to atomic resonance, and the nonclassical light generated is notably weaker than the input driving fields. Consequently, a fundamental challenge inherent in such scenarios is the derivation of linear and nonlinear susceptibilities governing the interplay of the involved electromagnetic (EM) fields.

In the realm of optical interactions, the computational landscape for determining susceptibilities becomes more intricate when dealing with scenarios involving multiple EM fields acting on the same atomic transition. In cases where only one EM field per atomic transition is implicated, established methods such as density-matrix formalism and master equations prove effective in calculating susceptibilities. Yet, as the complexity deepens, especially in the context of triphoton generation as examined in this study, novel strategies are necessitated.

Wen and colleagues have contributed a valuable approach (*32-34*) that facilitates precise susceptibility calculations, particularly relevant for generating entangled photon pairs. However, when applied to the triphoton generation investigated herein, the methodology faces heightened theoretical calculations. This complexity arises from the simultaneous presence of three EM fields–E_2, E_3, and E_{s2}–within a single atomic transition $|2\rangle - |4\rangle$ (as depicted in Fig. 1B in the main text). Ongoing efforts are dedicated to advancing the exact derivations using this method.

In the interim, we employ a "qualitative" technique–perturbation chain rule–to explore the optical response of atomic vapor in the context of triphoton emission and its associated optical attributes. This qualitative approach has found application in analogous atomic systems with comparable energy-level structures, yielding results that align comparably. Moreover, it has been employed to analyze light-atom interaction (*40-45*) in the context of six-wave mixing (SWM) in the stimulated emission regime. As elucidated below, while *the derived qualitative optical response results* may not align seamlessly with the experimental data, they do furnish a reasonable framework for comprehending the observed triphoton behaviors.

The foundation of this qualitative approach is firmly grounded in perturbation theory, which prioritizes the dressing steady states while overlooking the transient propagation influence. The initial stage involves perturbative examination of the SWM process, leveraging the framework of weak-field approximation. Subsequently, the dressing perturbation strategy is invoked, establishing a set of strongly coupled equations driven by the strong fields. This framework thus facilitates the determination of density-matrix elements via the perturbation chain rule.

Following the methodology akin to that expounded in Refs. (*40-45*), it is revealed that the fifth-order nonlinear susceptibility $\chi^{(5)}$ can be approximately attained from the ensuing perturbation chain:

$$\rho_{11}^{(0)} \xrightarrow{\omega_1} \rho_{31}^{(1)} \overset{\omega_{S1}}{-} \rho_{21}^{(2)} \xrightarrow{\omega_2} \rho_{41}^{(3)} \overset{\omega_{S2}}{-} \rho_{11}^{(4)} \xrightarrow{\omega_3} \rho_{41}^{(5)}, \tag{S1}$$

where ω_1, ω_2 and ω_3 denote the frequencies of the three input lasers, while ω_{S1}, ω_{S2} and ω_{S3} represent the frequencies of the generated triphotons. By solving the series of density-matrix

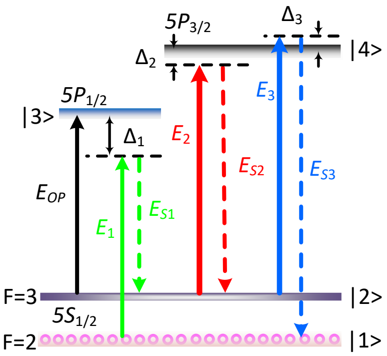

Figure S1: **Energy-level diagram of hot ^{85}Rb atoms illustrating direct time-energy-entangled W-class triphoton generation.** This four-level triple-Λ-type atomic configuration features two ground states $|1\rangle$ and $|2\rangle$, as well as two excited states $|3\rangle$ and $4\rangle$. Initial atomic population is established in state $|1\rangle$. To prevent residual atomic population in $|2\rangle$, an additional resonant optical pumping beam E_{OP} is introduced for the atomic transition $|2\rangle \leftrightarrow |3\rangle$. A weak cw pump laser E_1 is directed towards $|1\rangle \rightarrow |3\rangle$ with a large, fixed red frequency detuning Δ_1. Meanwhile, another two strong cw control fields, E_2 and E_3, are concurrently applied to the same atomic transition $|2\rangle \rightarrow |4\rangle$, but with different frequency detunings Δ_2 and Δ_3. By adhering to the required phase-matching conditions, the spontaneous six-wave mixing (SSWM) process is facilitated, enabling the direct and efficient emission of continuous-mode time-energy-entangled W-type triphotons—E_{S1}, E_{S2} and E_{S3}—from their respective atomic transitions. This emission process is visually depicted in the diagram.

equations, one can deduce the density-matrix elements $\rho_{11}^{(0)}, \rho_{31}^{(1)}, \ldots, \rho_{41}^{(5)}$ in Eq. (S1) through a stepwise progression. Given the nature of atomic vapor, the incorporation of Doppler broadening effects become imperative. After some lengthy calculations, we have finally derived the fifth-order nonlinear susceptibility characterizing the light-atom interaction, as displayed in Fig. S1. This susceptibility adopts the following form:

$$\chi^{(5)}(\delta_2,\delta_3) = \int_{-\infty}^{\infty} dv \frac{2N\mu_{13}\mu_{24}\mu_{23}\mu_{14}^3 f(v)}{\varepsilon_0\hbar^5\left\{\begin{matrix}(\Gamma_{31}+i\Delta_{D1})[(\Gamma_{21}+iW_{D-}\delta_2+iW_{D+}\delta_3)(\Gamma_{41}+iW_{D-}\delta_2+iW_{D+}\delta_3+i\Delta_{D2})+|\Omega_2|^2] \\ \times[(\Gamma_{11}+iW_{D+}\delta_3)(\Gamma_{41}+iW_{D+}\delta_3+i\Delta_{D3})+|\Omega_3|^2]\end{matrix}\right\}}. \quad \text{(S2)}$$

Here, $f(v) = \sqrt{\frac{m_{\mathrm{Rb}}}{2\pi k_B T}} e^{-\frac{m_{\mathrm{Rb}} v^2}{2k_B T}}$ is the Maxwell-Boltzmann velocity distribution of Rb atoms in thermal motion, where m_{Rb} is the mass of the Rb atom, k_B is the Boltzmann constant, T is the vapor temperature, and v is the atomic kinetic velocity; N denotes the atomic density; μ_{ij} $(i,j = 1,2,3,4)$ represents the electric dipole matrix element for the atomic transition $|i\rangle \rightarrow |j\rangle$; ε_0 stands for the vacuum permittivity; Γ_{ij} is the decay or decoherence rate between levels $|i\rangle$ and $|j\rangle$; $\Delta_{D1} = \Delta_1 + v\omega_{31}/c$, $\Delta_{D2} = \Delta_2 - v\omega_{42}/c$, and $\Delta_{D3} = \Delta_3 + v\omega_{42}/c$ are associated with the frequency detunings $\Delta_1 = \omega_{31} - \omega_1$, $\Delta_2 = \omega_{42} - \omega_2$, and $\Delta_3 = \omega_{42} - \omega_3$, where ω_{ij} is the frequency difference between $|i\rangle$ and $|j\rangle$; $W_{D\pm} = 1 \pm v/c$ depends on atomic velocity with c the speed of light in vacuum; Ω_2 and Ω_3 are the Rabi frequencies; δ_2 and δ_3 define the spectral distributions with respect to the central frequencies of the emitted E_{S2} and E_{S3} photons, respectively. Additionally, it's important to note that owing to the energy conversation in SSWM, the triggers for these two photons require the detection of the output E_{S1} photon at frequency $\omega_{S1} = \omega_1 + \omega_2 + \omega_3 - \omega_{S2} - \omega_{S3}$. This alternatively implies that the spectral distributions of the entangled three-photon state need to satisfy the condition $\delta_1 + \delta_2 + \delta_3 = 0$.

When $T = 80°\mathrm{C}$, the Doppler width is estimated to be approximately Δ_D= 555 MHz, orders of magnitude larger than the Rb natural linewidth. The atomic density is given by $N = 1.2 \times 10^{11}$

cm^{-3}. The optical depth (OD), calculated as $OD = N\sigma_{41}L$, amounts to 4.6, where $\sigma_{41} = \frac{\omega_{41}|\mu_{14}|^2}{2\varepsilon_0\hbar c\Gamma_{41}\Delta_D} = 3\pi N\Gamma_{41}c^2L/2\Delta_D\omega_{41}^2$ stands for the on-resonance absorption cross-section of the transition $|1\rangle \to |4\rangle$. At a higher temperature $T = 115°\text{C}$, the OD grows significantly to the value of 45.7.

In accordance with our previous theoretical investigations (*9*, *28*, *32-34*, *40-50*), the temporal correlations inherent in the triphoton generation are impacted by two primary factors: the spectral profile of the fifth-order nonlinear susceptibility $\chi^{(5)}$, as provided by Eq. (S2), and the longitudinal phase-mismatch function, which will be discussed in subsequent sessions. With this premise, we initiate our examination by delving into the at the structure of $\chi^{(5)}$.

Similar to our earlier analyses (*9*, *28*, *32-34*, *40-50*), the resonances originating from the denominator of $\chi^{(5)}$ in Eq. (S2) are centrally located around $\delta_{1\pm} = \left(\Delta_{D2} \pm \Omega_{E_2}\right)/2(1 - \frac{v}{c})$, $\delta_{2\pm\pm} = \left(\Delta_{D3} - \Delta_{D2} \pm \Omega_{E_2} \pm \Omega_{E_3}\right)/2(1 + \frac{v}{c})$, and $\delta_{3\pm} = \left(-\Delta_{D3} \pm \Omega_{E_3}\right)/2(1 - \frac{v}{c})$. Here, the effective Rabi frequencies are redefined as $\Omega_{E_2} = \sqrt{\Delta_{D2}{}^2 + 4|\Omega_2|^2 + 4\Gamma_{21}\Gamma_{41}}$ and $\Omega_{E_3} = \sqrt{\Delta_{D3}{}^2 + 4|\Omega_3|^2 + 4\Gamma_{11}\Gamma_{41}}$, with Ω_2 and Ω_3 being the original Rabi frequencies of the E_2 and E_3 fields, respectively. Notably, the effective linewidths of these resonances are determined by the imaginary components of the denominator. These linewidths, $\Gamma_{\delta_2} = \frac{\Gamma_{21}+\Gamma_{41}}{2} + \frac{\Gamma_{21}\Delta_{D2}}{\Delta_{D2}+\Omega_{E_2}}$ and $\Gamma_{\delta_3} = \frac{\Gamma_{11}+\Gamma_{41}}{2} + \frac{\Gamma_{11}\Delta_{D3}}{\Delta_{D3}+\Omega_{E_3}}$, are responsible for setting the temporal correlation lengths between generated triphotons. Importantly, these resonance centers and effective linewidths are both contingent on the velocity of the atomic motion, and are thus influenced by the Doppler broadening effect.

By analyzing the calculated $\delta_{1\pm}$, $\delta_{2\pm\pm}$ and $\delta_{3\pm}$, it is anticipated that there will generally exist four sets of indistinguishable SSWM processes, facilitating the production of time-energy-entangled W-triphotons. As an illustrative instance, Fig. S2 visually presents the behavior of $\chi^{(5)}$ across different scenarios. A keen observation reveals that, upon velocity integration, for cases with low OD, four distinct resonances will typically manifest (Figs. S2A and S2B); whereas for high OD values, the possibility arises to coalesce four resonances into two (Fig. S2C).

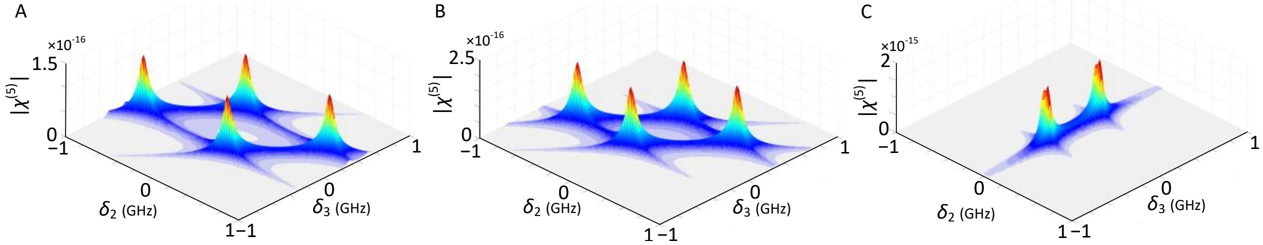

Figure S2: **Exemplary visualization of the fifth-order nonlinear susceptibility $\chi^{(5)}$, computed by the perturbation-chain rule, across different parameter configurations.** (**A**) $\chi^{(5)}$ corresponding to **Fig. 2A** of the main text, utilizing the following simulation parameters: $\Gamma_{31} = \Gamma_{41} = 2\pi \times 6$ MHz, $\Gamma_{11} = \Gamma_{22} = 0.4 \times \Gamma_{41}$, $\Gamma_{21} = 0.2 \times \Gamma_{41}$, $\Delta_1 = -2\text{GHz}$, $\Delta_2 = -150\text{MHz}$, $\Delta_3 = 50\text{MHz}$, $OD = 4.6$, $\Omega_1 = 300$ MHz, $\Omega_2 = 870$ MHz, and $\Omega_3 = 533$ MHz. Input laser powers are set at $P_1 = 4$ mW, $P_2 = 40$ mW, and $P_3 = 15$ mW. (**B**) $\chi^{(5)}$ corresponding to **Fig. 3A** of the main text, utilizing the same simulation parameters as (**A**), with the exception of $\Omega_2 = 533$ MHz and input power $P_2 = 15$ mW. (**C**) $\chi^{(5)}$ corresponding to **Fig. 3D** of the main text, employing the same simulation parameters as (**B**), except for $OD = 45.7$.

Qualitative Derivations of Linear Susceptibilities χ

Apart from the resonance linewidths governed by $\chi^{(5)}$, the temporal correlation of triphotons is also dependent on dispersion, which stems from the linear optical response. By applying the appropriate perturbation chain rule, after some calculations we obtain the individual linear susceptibilities of the new E_{S1}, E_{S2} and E_{S3} fields, yielding the following expressions:

$$\chi_{S1} \approx 0, \tag{S3}$$

$$\chi_{S2} = \int f(v) \frac{-i4N\mu_{24}^2\left((1-\frac{v}{c})\delta_2+i\Gamma_{22}\right)}{\varepsilon_0\hbar\left[4\left((1-\frac{v}{c})\delta_2-\Delta_{D2}+i\Gamma_{42}\right)\left((1-\frac{v}{c})\delta_2+i\Gamma_{22}\right)+|\Omega_2|^2\right]} dv, \tag{S4}$$

$$\chi_{S3} = \int f(v) \frac{-i4N\mu_{14}^2\left((1+\frac{v}{c})\delta_3+i\Gamma_{11}\right)}{\varepsilon_0\hbar\left[4\left((1+\frac{v}{c})\delta_3-\Delta_{D3}+i\Gamma_{41}\right)\left((1+\frac{v}{c})\delta_3+i\Gamma_{11}\right)+|\Omega_3|^2\right]} dv. \tag{S5}$$

Eq. (S3) is amply substantiated by the utilization of a weak input E_1 beam coupled with an exceedingly large red detuning $\Delta_1 = -2$ GHz from the transition $|1\rangle \rightarrow |2\rangle$. This outcome indicates that the group velocity of the E_{S1} photons closely approximates the speed of light in vacuum, c. To enhance understanding, Fig. S3 encompasses numerical simulations of χ_{S2} and χ_{S3}, elucidating the features of their profiles. Consequently, the group velocities experienced by the E_{S2} and E_{S3} photons are routinely derived using the formula:

$$v_{S2} = \left(\frac{dk_{S2}}{d\omega}\right)^{-1} = \frac{c}{1+\delta_2(\frac{dn_{S2}}{d\delta_2})}, \tag{S6}$$

$$v_{S3} = \left(\frac{dk_{S3}}{d\omega}\right)^{-1} = \frac{c}{1+\delta_3(\frac{dn_{S3}}{d\delta_3})}, \tag{S7}$$

where $n_{S2} = \sqrt{1+\mathrm{Re}[\chi_{S2}]}$ and $n_{S3} = \sqrt{1+\mathrm{Re}[\chi_{S3}]}$ are refractive indices. The imaginary parts of χ_{S2} and χ_{S3} ascribe the linear Raman gain or absorption undergone by the E_{S2} and E_{S3} photons during their traversal through the medium. Armed with this insight, the computation of the longitudinal phase mismatch in the SSWM process becomes apparent. This mismatch is defined as

$$\Delta k(\delta_2, \delta_3) = k_{S1} - k_{S2} + k_{S3} - k_1 + k_2 - k_3, \tag{S8}$$

where $k_j = \overline{k_j} + \frac{\omega}{v_j}$ ($j = 1,2,3,S1,S2,S3$), and $\overline{k_j}$ denotes the central wavenumber. Equation (S8) underscores the inherent spectral width of the generated triphoton state, thereby serving as a natural determinant for the temporal coherence time due to the influence of light propagation within the atomic vapor.

To offer insights into the behavior of χ_{S2} and χ_{S3}, we present an illustrative example in Fig. S3, showcasing their real and imaginary components post the Doppler integration. As one can see, χ_{S2} and χ_{S3} typically exhibit two resonance structures, as visualized in Figs. S3A–D. This divergence from the four resonances observed in $\chi^{(5)}$ (depicted in Figs. S2A and S2B) can be attributed to the qualitative model employed for the calculation of linear (and nonlinear) susceptibilities. We are presently engaged in refining this understanding by undertaking precise theoretical computations of both linear and nonlinear optical responses, leveraging the accurate model (*32-34*) pioneered by Wen *et al*. The outcomes of this ongoing effort are slated for publication in an upcoming venue. Meantime, we are open to the emergence of alternative theories from the community, as the associated mathematics is highly complex. We enthusiastically welcome the development of new

theories that can accurately characterize these optical responses. We are optimistic that this complexity presents an opportunity for our work to inspire novel theoretical advancements. Unlike previous protocols that comfortably fit within the existing theoretical framework, our approach challenges it and encourages fresh theoretical development.

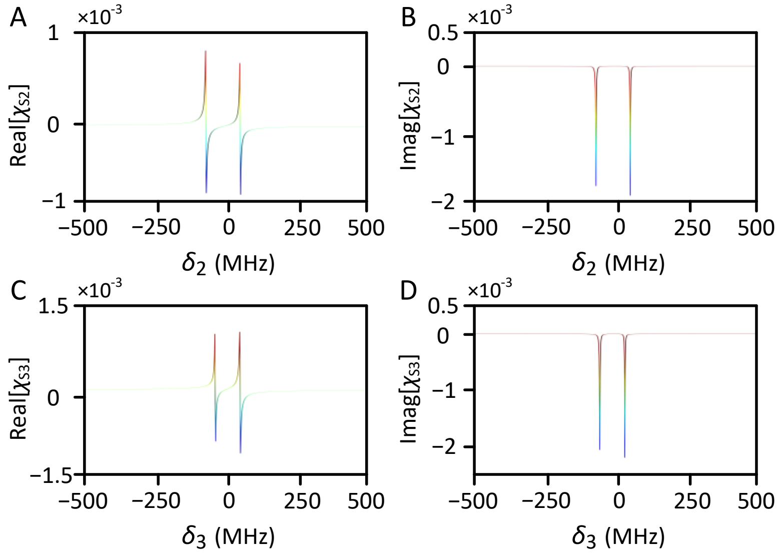

Figure S3: **Representative illustrations of linear susceptibilities χ_{S2} and χ_{S3} computed by the perturbation chain rule.** The parameters involved remain consistent with those employed in **Fig. S2B**. (**A & B**) Display of the real and imaginary parts of χ_{S2}. (**C & D**) Depiction of the real and imaging components of χ_{S3}.

Derivation of the Triphoton State $|\Psi\rangle$

To calculate the resultant three-photon state stemming from the SSWM process at the output surface of the medium, we shall work in the Schrödinger picture (*51, 52*). We commence with the following effective interaction Hamiltonian,

$$H = \int_0^L dz\varepsilon_0 \chi^{(5)} E_1^{(+)} E_2^{(+)} E_3^{(+)} E_{S1}^{(-)} E_{S2}^{(-)} E_{S3}^{(-)} + H.c., \tag{S9}$$

where $H.c.$ means the Hermitian conjugate. Here, the generated E_{S1}, E_{S2} and E_{S3} photons are described by the quantized electric fields,

$$E_{Sj}^{(+)} = \sum_{k_{Sj}} E_{Sj} a_j e^{i(k_{Sj}z - \omega_{Sj}t)}, \tag{S10}$$

where a_j symbolizes the annihilation operator for the mode with the wavenumber k_{Sj} and angular frequency ω_{Sj}. Additionally, $E_{Sj} = i\sqrt{\hbar\omega_{Sj}/2\varepsilon_0 n_{Sj}^2 L}$. On the other hand, the three input continuous-wave (cw) lasers E_1, E_2, and E_3 are taken as classical plane waves,

$$E_1^{(+)} = E_1 e^{i(k_1 z - \omega_1 t)},\ E_2^{(+)} = E_2 e^{i(-k_2 z - \omega_2 t)},\ \text{and}\ E_3^{(+)} = E_3 e^{i(k_3 z - \omega_3 t)}. \tag{S11}$$

The state vector of the triphotons can then be ascertained through first-order perturbation theory (*9, 28, 32-34, 40-52*):

$$|\Psi\rangle = \frac{-i}{\hbar}\int_{-\infty}^{+\infty} dtH\,|0\rangle, \tag{S12}$$

with $|0\rangle$ being the initial vacuum state. By applying Eqs. (S9)–(S12) and ignoring the vacuum term that has no effect in photon clicks, the triphoton state (S12) can be formulated as:

$$|\Psi\rangle = \sum_{k_{S1}} \sum_{k_{S2}} \sum_{k_{S3}} F(k_{S1}, k_{S2}, k_{S3})\, a^{\dagger}_{k_{S1}} a^{\dagger}_{k_{S2}} a^{\dagger}_{k_{S3}} |0\rangle, \tag{S13}$$

where the three-photon spectral function F is defined as

$$F(k_{S1}, k_{S2}, k_{S3}) = A\chi^{(5)}\Phi(\Delta kL)\delta(\omega_1 + \omega_2 + \omega_3 - \omega_{S1} - \omega_{S2} - \omega_{S3}), \tag{S14}$$

with A being a grouped constant. In Eq. (S14), the Dirac δ function comes from the time integral in the steady-state approximation, ensuring the energy conservation in the SSWM process. From the perspective of atomic population, this energy conservation implies that after a triphoton generation cycle, the population returns to its initial ground state $|1\rangle$. $\Phi(\Delta kL)$ is the so-called longitudinal phase-mismatch function, taking the form of:

$$\Phi(\Delta kL) = \frac{1 - e^{-i\Delta kL}}{i\Delta kL} = \mathrm{sinc}\left(\frac{\Delta kL}{2}\right) e^{-i\Delta kL/2}. \tag{S15}$$

Due to the Doppler effect in $\chi^{(5)}$ and Δk, an exact analytical expression for the triphoton state (S13) becomes challenging. Instead, hereafter we will rely on numerical analysis to unveil the triphoton properties.

Derivations of Temporal Correlations of W Triphotons

The optical properties of the W-type triphotons can be comprehensively understood by examining their photon statistics through photon-counting measurements. Consequently, we delve into the temporal correlation of triphotons by evaluating the Glauber second-order and third-order correlation functions (*9, 14, 53, 54*). This exploration then prompts us to carry out the analysis of conditioned two~photon coincidence counts and three-photon coincidence counts.

In line with the experimental setup illustrated in Fig. 1 of the main text, the average triphoton coincidence counting rate is expressed as:

$$R_3 = \lim_{T\to\infty} \frac{1}{T} \int_0^T dt_1 \int_0^T dt_2 \int_0^T dt_3 \langle\Psi|\, E^{(-)}_{S1}(\tau_1) E^{(-)}_{S2}(\tau_2) E^{(-)}_{S3}(\tau_3) E^{(+)}_{S3}(\tau_3) E^{(+)}_{S2}(\tau_2) E^{(+)}_{S1}(\tau_1) |\Psi\rangle, \tag{S16}$$

and the conditional two~photon coincidence counting rate is:

$$R_2 = \lim_{T\to\infty} \frac{1}{T} \int_0^T dt_1 \int_0^T dt_2\, \langle\Psi| E^{(-)}_{S2}(\tau_2) E^{(-)}_{S3}(\tau_3) E^{(+)}_{S3}(\tau_3) E^{(+)}_{S2}(\tau_2) |\Psi\rangle, \tag{S17}$$

assuming, for instance, that the E_{s1} photons are traced away. In Eqs. (S16) and (S17), $E^{(+)}_{Sj}(\tau_j)$ ($j = 1,2,3$) is the positive frequency part of the free-space electric field evaluated at the spatial coordinate r_j of the jth detector alongside with its trigger (or click) time t_j, with $\tau_j = t_j - r_j/c$. For simplicity, we consider the efficiencies of all involved single-photon detectors to be unity. In addition, given that the narrow bandwidths (less than GHz) of the triphotons in question are comparable to or smaller than the spectral resolving width of the utilized single-photon detectors in our experiment, we can simplify Eqs. (S16) and (17) to:

$$R_3 = \left|\langle 0| E^{(+)}_{S3}(\tau_3) E^{(+)}_{S2}(\tau_2) E^{(+)}_{S1}(\tau_1) |\Psi\rangle\right|^2 = |A_3(\tau_1, \tau_2, \tau_3)|^2, \tag{S18}$$

$$R_2 = \sum_{k_{S1}} \left|\langle 0| a_{k_{S1}} E^{(+)}_{S3}(\tau_3) E^{(+)}_{S2}(\tau_2) |\Psi\rangle\right|^2 = \sum_{k_{S1}} |A_2(\tau_2, \tau_3)|^2, \tag{S19}$$

where $A_3(\tau)$ is often referred to as the three-photon amplitude or triphoton waveform. Notably, $A_2(\tau)$ also represents the three-photon amplitude, even though one subsystem is not detected in the experiment. It's essential to emphasize that both $A_3(\tau)$ and $A_2(\tau)$ are defined with reference

to photon detections. By plugging Eq. (S13) into Eq. (S18), we attain:

$$A_3(\tau_1,\tau_2,\tau_3) = A_3 \sum_{k_{S1}} \sum_{k_{S2}} \sum_{k_{S3}} e^{-i(\omega_{S1}\tau_1+\omega_{S2}\tau_2+\omega_{S3}\tau_3)} F(k_{S1},k_{S2},k_{S3}), \tag{S20}$$

where all slowly varying terms and constants have been absorbed into A_3. Similarly, by substituting Eq. (S13) into Eq. (S19), we get:

$$A_2(\tau_2,\tau_3) = A_2 \sum_{k_{S2}} \sum_{k_{S3}} e^{-i(\omega_{S2}\tau_2+\omega_{S3}\tau_3)} F(k_{S1},k_{S2},k_{S3}), \tag{S21}$$

where again, all the slowly varying terms and constants have been grouped into A_2. Furthermore, to evaluate the Dirac δ function in F (S14), we replace the summation over wavenumber with an angular frequency integral as usual,

$$\sum_{k_{Sj}} \to \frac{L}{2\pi}\int d\omega_{Sj}\frac{dk_{Sj}}{d\omega_{Sj}} = \frac{L}{2\pi}\int \frac{d\omega_{Sj}}{v_{Sj}}. \tag{S22}$$

Using Eqs. (S13) and (S22), the three-photon amplitude (S20) becomes

$$A_2(\tau_{21},\tau_{31}) = A_3 \int\int d\delta_2\, d\delta_3 \chi^{(5)}(\delta_2,\delta_3)\mathrm{sinc}\left[\frac{\Delta k(\delta_2,\delta_3)L}{2}\right] e^{-i\delta_2(\tau_{21}+L/2v_{S2})} e^{-i\delta_3(\tau_{31}+L/2v_{S3})}. \tag{S23}$$

The three-photon coincidence counting rate (S18) is simply modulus squared of $A_3(\tau_{21},\tau_{31})$, i.e., $R_3 = |A_3(\tau_{21},\tau_{31})|^2$, which gives complex three-dimensional temporal correlations. Unlike the biphoton case in traditional studies, understanding the temporal correlations among the three photons within the same triphoton pair presents intellectual challenges, as the theoretical framework for biphoton physics does not directly apply. To address this issue, one can explore temporal correlations by analyzing triphoton coincidences along different slicing directions. For instance, in the main text, we examine temporal correlations by selecting $\tau_{21} + \tau_{31} = 15$ ns (Fig. 2E) to highlight the significance of intricate 3D triphoton temporal correlations.

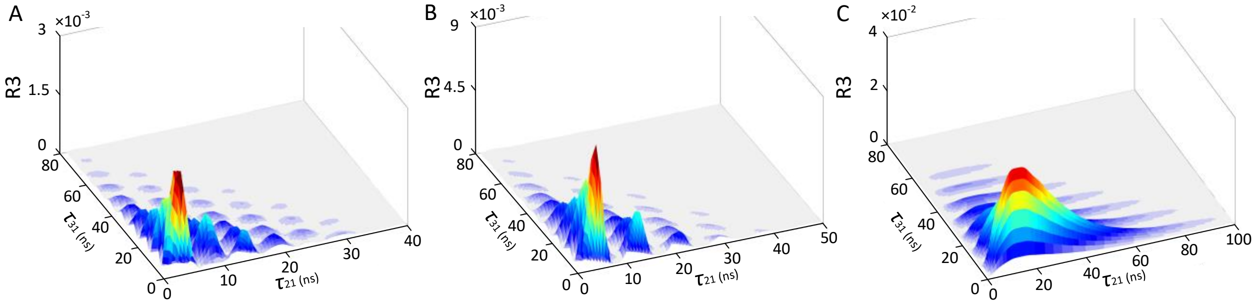

Figure S4: **Theoretical simulations of triphoton threefold coincidence counting rates R_3 using the optical responses computed by the perturbation chain rule.** (**A**) R_3 associated with **Fig. 2A** in the main text, employing identical parameters to those featured in **Fig. S2A**. (**B**) R_3 corresponding to **Fig. 3A** in the main text, utilizing the same parameters as those in **Fig. S2B**. (**C**) R_3 related to **Fig. 3D** in the main text, using the same parameters as those in **Fig. S2C**.

From Eq. (S23), it is evident that the three-photon amplitude $A_3(\tau_{21},\tau_{31})$ is the convolution of the fifth-order nonlinear susceptibility $\chi^{(5)}(\delta_2,\delta_3)$ and the longitudinal phase-mismatch function $\Phi(\Delta kL)$. Physically, this implies that the triphoton temporal coherence is jointly determined by these two factors. As a consequence, we anticipate the appearance of two distinct regions in three-photon temporal correlation measurements, characterized by the damped Rabi oscillation regime dominated by $\chi^{(5)}$ and the group-delay regime dominated by $\Phi(\Delta kL)$. These regions have been explored in the experiment, and the recorded data are presented in Figs. 2–4 of the main text, as

well as in Supplementary Figs. S11 and S12 (below). For qualitative comparison, Fig. S4 provides the corresponding theoretical simulations. It is apparent that both Figs. S4A and S4B exhibit the three-photon coincidence counts in the damped Rabi oscillation regime, while Fig. 4C displays the case in the group-delay region, qualitatively explaining the experimental observations in Figs. 2A, 3A, and 3D of the main text.

Similarly, we can demonstrate that the conditioned two~photon coincidence counting rate can be computed as:

$$R_2(\tau_{23}) = R_2 \int d\delta_3 \left| \int d\delta_2\, \chi^{(5)}(\delta_2, \delta_3) \mathrm{sinc}\left[\frac{\Delta k(\delta_2,\delta_3)L}{2}\right] e^{-i\delta_2(\tau_{23}+L/2v_{S2})} \right|^2, \quad \text{(S24)}$$

where $\tau_{23} = \tau_2 - \tau_3$ and R_2 is a grouped constant. As evident from Eq. (S24), $R_2(\tau_{23})$ is a function of τ_{23}, indicating the presence of partial entanglement between the remaining E_{S2} and E_{S3} photons after tracing away the E_{S1} photon. This unequivocally signifies the tripartite W-class property.

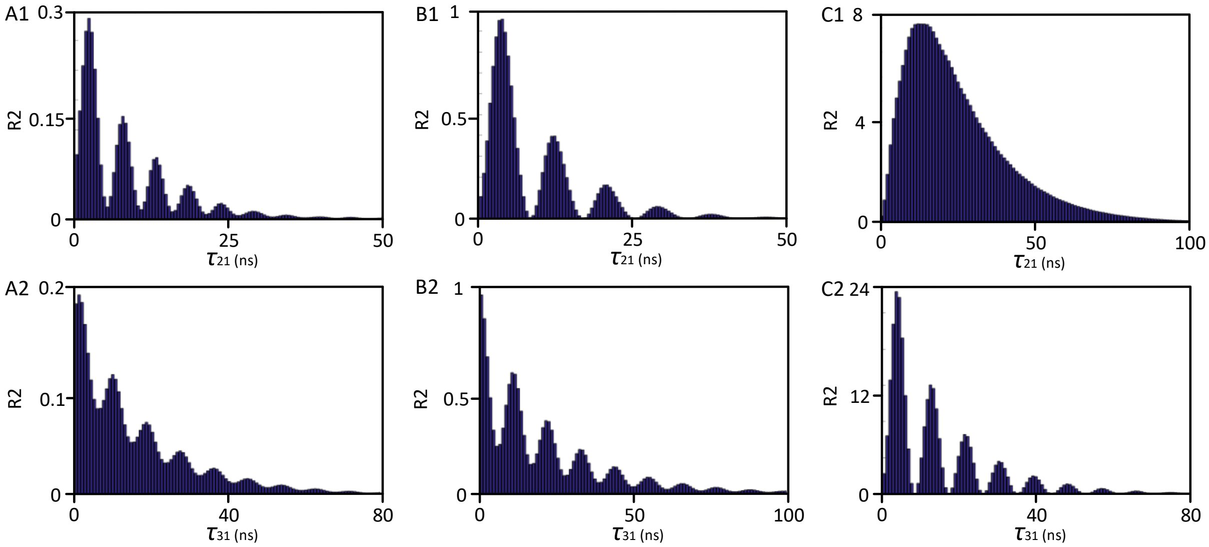

Figure S5: **Theoretical simulations of conditional two~photon coincidence counting rates R_2 for Fig. S4.** (**A1**) R_2 achieved by tracing away the E_{S3}-photons in **Fig. S4A**. (**A2**) R_2 attained by tracing away the E_{S2}-photons in **Fig. S4A**. (**B1**) R_2 acquired by tracing away the E_{S3}-photons in **Fig. S4B**. (**B2**) R_2 acquired by tracing away the E_{S2}-photons in **Fig. S4B**. (**C1**) R_2 achieved by tracing away the E_{S3}-photons in **Fig. S4C**. (**C2**) R_2 attained by tracing away the E_{S2}-photons in **Fig. S4C**.

In Eq. (S24), the second integral inside the modulus squared is a convolution between $\chi^{(5)}$ and $\Phi(\Delta kL)$. Similarly, the functional profile of $R_2(\tau_{23})$ is in general determined by both factors. However, if one of these factors predominates, $R_2(\tau_{23})$ will showcase two distinctive scenarios: the damped Rabi oscillation regime and the group-delay regime. Other configurations for conditional two~photon coincidence counts can be calculated using the same logic. Here, we refrain from reiterating those computations and leave them as an exercise for the reader. In Figs. 2C and 2D, as well as Figs. 3B, 3C, 3E, and 3F in the main text, we present examples of such measured conditional two~photon coincidence counts. For qualitative comparison, Fig. S5 provides the corresponding theoretical simulations. It is evident that our theoretical framework aligns qualitatively with the experimental results. However, when comparing qualitative calculations with actual experimental data, significant differences become apparent. Wen and his colleagues have recently advanced in precise calculations of linear and nonlinear optical responses

using the harmonic expansion method he developed. Initial calculations indicate promising agreement between theory and experiment. Further verification is underway, and the detailed results will be published in a separate publication.

Triphoton W State Entangled in Other Degrees of Freedom

While the primary focus of this study revolves around time-energy-entangled W triphotons, it is important to acknowledge that these W-class triphotons can also be readily entangled in other degrees of freedom, encompassing space-momentum, polarization, and orbital angular momentum. In other words, our work uniquely provides a dependable genuine W-class triphoton source, capable of generating a range of three-photon W states entangled across diverse degrees of freedom without involving additional interferometry setups or postselection. For example, our triphoton source can effortlessly yield triphotons entangled in space or momentum due to phase matching. Our source can also directly produce polarization-based W triphotons, without necessitating an interferometer, by inputting three linearly polarized cw lasers. The heightened SSWM process facilitated by atomic coherence enables the exploration of diverse forms of three-photon entanglement based on different degrees of freedom. This would be challenging or even unattainable using previously proposed schemes or methods.

Furthermore, our system exhibits the capability to generate triphoton hyperentangled states, entangling more than one degree of freedom of light. This introduces a significant technical challenge for any multiphoton generation platform reported thus far. While the system layouts and theoretical calculations concerning these triphoton entangled states are beyond the scope of this work, they will be elaborated upon in the forthcoming discussions.

Significantly, triphotons entangled in distinct degrees of freedom offer unique opportunities for quantum technological applications. For instance, the W-type triphotons endowed with spatial correlations (*14*) can be harnessed for quantum imaging and remoting sensing (*54*), enabling sub-Rayleigh superresolution that is both beyond the capabilities of biphotons (or entangled photon pairs) and classical light. This solidifies the fundamentally quantum nature of these phenomena and their potential to redefine quantum technologies.

Beyond the primary focus on the continuous-mode scenario explored in this study, our system seamlessly extends its capabilities to encompass the continuous variable (CV) regime. Within this framework, the generation of non-Gaussian (Wigner function) tripartite states becomes a tangible achievement, facilitating their utilization across a spectrum of CV-based quantum information and computing protocols (*55*). This underscored adaptability and versatility inherent in our triphoton source stand as distinguishing features, setting it apart from many preceding methodologies overviewed in the main text.

Addressing Misconceptions: Clearing Up Common Misunderstandings

In what follows, we would like to clarify some misconceptions prevalent in studies related to multiphoton generation. Through careful examination of the existing literature, we are aware of several prevalent misunderstandings in the realm of multiphoton entanglement generation:

- *Equating multiphoton source with specific multiphoton state*. A prevalent misconception arises when the community conflates an “entangled multiphoton source” with “the realization of a specific entangled state.” It's crucial to discern the fundamental distinction between these two concepts. The former encompasses the latter comprehensively, while the latter represents only a singular instance. Our work’s significance lies in introducing a reliable genuine W-class triphoton source, capable of generating diverse three-photon W states entangled across various degrees of freedom—eliminating the need for additional interferometry and postselection. Although we demonstrated time-energy triphoton entanglement, our source effortlessly

produces triphotons entangled in space or momentum due to phase matching. This starkly contrasts with most prior multiphoton state demonstrations, which only achieve detection potential without acting as dependable multiphoton sources. Our approach, in contrast, ensures exclusive production of desired states due to the unique phase matching, guaranteeing confident, high-purity, and high-fidelity triphoton generation.

- *Comparing incompatible classes*. Recognizing the essential incongruity between the GHZ and W classes is paramount. This divergence underscores the importance of contextualizing the superiority of one class over the other within specific problems or applications. Without this contextual framework, any comparison lacks substantive relevance, rendering it incapable of enriching our understanding of multipartite entanglement. Furthermore, this inherent incompatibility leads to an intriguing consequence: any endeavor to transform a given class into its opposite counterpart demands the incorporation of supplementary interferometric setups and postselection measurements. Failing to do so renders such conversions unattainable.
- *Multiphoton production utilizing cascaded SPDCs/SFWMs*. the utilization of cascaded SPDCs or SFWMs for generating time-energy triphotons has demonstrated constrained dependability and suboptimal fidelity. This issue stems from the necessity of preserving over thousands of leftover single photons resulting from the primary SPDC or SFWM process, awaiting the emergence of a singular pair from the subsequent process. As a consequence, in the absence of sophisticated detection systems, ensuring consistent production of a solitary triphoton entity remains elusive. Using a neutral density filter cannot guarantee that the retained photon is the one exactly associated with the secondary biphoton process. This inherent limitation and drawback render the feasibility of these methodologies ineffectual for establishing a dependable and authentic triphoton source.
- *Comprehending multiphoton entanglement with biphoton knowledge*. While our physics research typically begins by comprehending low-dimensional and simple scenarios before attempting to generalize to high-dimensional and complex situations, it is crucial to be mindful of the limitations of this approach when studying multi-photon entanglement. One significant challenge, as repeatedly emphasized, arises from the fact that, unlike the biphoton case, the triphoton scenario involves two incompatible classes. Understanding these properties goes beyond the knowledge derived from the biphoton context.

It also comes to our attention that even though multiphoton polarization-based GHZ state (including the four-photon case) can be derived from SPDC or SFWM processes—given that these processes generate photons in pair—one must recognize that these multiphoton outputs arise from higher-order perturbation terms. To effectively detect such photon states, the construction of sophisticated detection systems becomes imperative in order to mitigate accidental counts stemming from dual photon pairs. Without effectively mitigating these prevalent photon trigger events originating from lower perturbations, the viability of establishing a reliable source using this scheme remains unattainable.

All in all, despite the multitude of protocols proposed over the last two decades for generating multiphoton entangled states, as comprehensively discussed in the main text, our perspective suggests that none of these protocols have matured into dependable multiphoton sources. This sentiment is rooted in the presence of inherent limitations and external challenges within these methodologies. Conversely, our devised scheme emerges as the most promising candidate to date for realizing a genuinely practical W-class triphoton source, bringing us notably closer to achieving this elusive goal.

II. Further Insights into Experimental Measurements and Data Processing

In the subsequent subsections, we would like to delve into the experimental measurements and data processing with greater depth. Additionally, we will present an extended collection of experimental data on triphoton coincidences, offering further evidence that the suggested SSWM process within coherent atomic ensembles efficiently produces genuine triphotons of exceptional quality and reliability. Notably, these supplementary findings, combined with the data presented in the main text, provide a comprehensive illustration of the source's versatility. This versatility holds the potential to unlock novel technological advancements that are currently beyond the reach of existing photon resources.

Possible Biphoton Processes

As outlined in the Methods section, a significant source of accidental coincidence noise in the three-photon correlation measurements mainly stem from the simultaneous occurrence of two pairs of biphotons, originating from distinct spontaneous four-wave mixing (SFWM) processes, detected by the single-photon detectors. Fortunately, these SFWMs exhibit differing phase matching conditions, deviating from the one pertinent to the SSWM process. Furthermore, the biphotons resulting from these SFWMs possess distinct central frequencies in contrast to those of the desired triphotons.

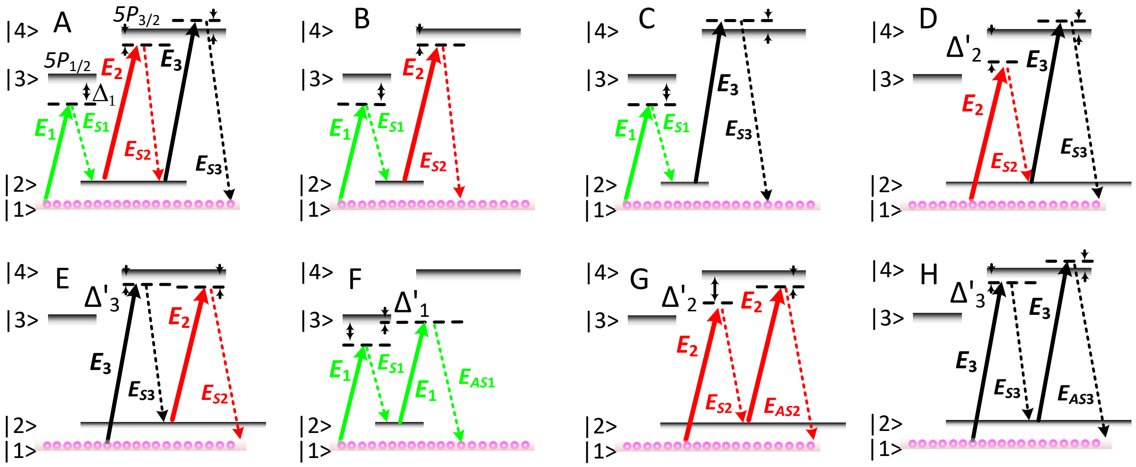

Figure S6: **Seven potential SFWM processes leading to accidental coincidences in three-photon coincidence counting measurement.** (**A**) Illustration of the atomic energy-level structure governing triphoton generation. (**B-H**) Seven distinct possible SFWM processes, each outlining scenarios where emitted biphotons might inadvertently contribute to accidental coincidences within the three-photon coincidence counts that are measured.

By meticulous manipulation of the phase matching conditions and the implementation of narrowband filters, the false trigger events from these biphotons can be effectively eliminated from the authentic triphoton coincidence counts. For a visual representation of these biphoton generation scenarios, Fig. S6 provides a schematic depiction of all possible SFWM processes. Leveraging the level structure, seven such SFWM processes have been identified and visually presented in Figs. S6B–H. It's worth noting that the biphotons originating from these SFWMs constitute the primary source of accidental coincidences impacting the actual measurements. In the Methods section, we have extensively expounded upon the potential combinations of these SFWM processes that could lead to error-triggering events.

While it is theoretically possible to generate entangled quadraphotons through higher-order nonlinear wave mixing processes, the likelihood of their emission remains considerably low. Consequently, they do not pose a significant noise source for triphoton detection. Given this

context, we will refrain from delving further into the discussion of entangled quadraphotons in this context.

Coincidence Counts obtained by Background Accidental Subtraction

Figures 2 and 3 in the main text showcase the recorded data alongside background accidental counts. In the corresponding Figs. S7 and S8, we present the same measured data after background accidental counts have been subtracted. A comparison between Figs. 2 and 3 and Fig. S7 and S8 underscores that the crucial characteristics remain well-preserved in both instances.

In Figs. S7C, S7D, S8B, S8C, and S8F, we have incorporated green and red dashed lines based on the measured data to highlight the oscillation periods referenced in the main text. By juxtaposing Figs. S7C, S7D, S8B, S8C, S8E, and S8F with Figs. S4A1–C2, we acknowledge that our qualitative optical response model can only furnish a qualitative interpretation of the experimental outcomes. Nonetheless, this approach effectively reveals fundamental features within the measurements.

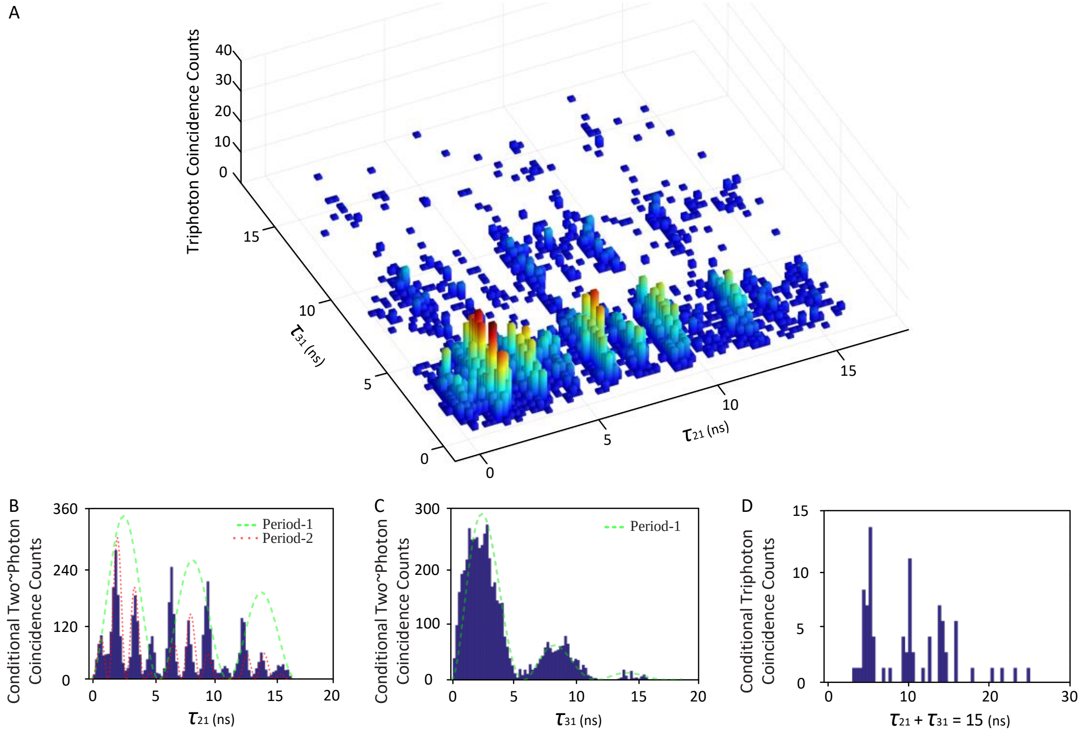

Figure S7: **Triphoton coincidence counts and conditioned two~photon & three-photon coincidence counts from Fig. 2 (main text), after background accidental removal.** (**A**) The measured data in **Fig. 2A** of the main text with background accidentals subtracted. In panels (**B & C**), periodic oscillations discussed in the main text are visualized using green and red dashed lines for the conditional two~photon temporal coincidences, as reported in **Figs. 2C** and **2D** of the main text, after subtracting background accidentals. (**D**) The measured data in **Fig. 2E** of the main text with background accidentals removal.

For a more comprehensive grasp of both conditional two~photon coincidences and conditional three-photon coincidences, we have extended our analysis beyond Figs. 2A, 3A, and 3D in the main text. By carefully removing the corresponding background accidental counts and exploring varied scenarios, we gain deeper insights. Figure S9 serves as an illustrative example of this processed experimental data, meticulously organized to adhere to specific conditions. Within these figures, it becomes evident that the coherence length of the residual temporal correlation for the two~photon scenario is not fixed; rather, it varies contingent upon the specific measurement

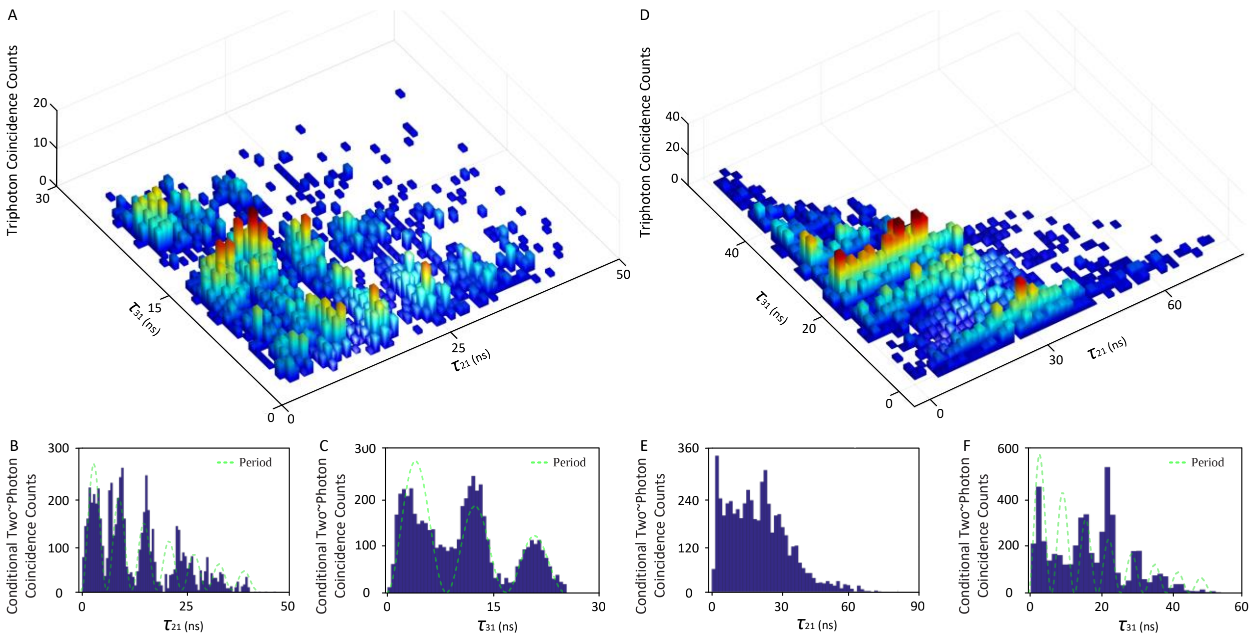

Figure S8: **Triphoton coincidence counts and conditioned two~photon & three-photon coincidence counts from Fig. 3 (main text), after background accidental subtraction.** Panels (**A–F**) correspond to the measured data reported in **Figs. 3A-3F** of the main text, with background accidental coincidences removed. In panels (**B, C & F**), the presence of periodic oscillations, as discussed in the main text, is visually highlighted through the use of green dashed lines for comprehension.

conditions. This variability similarly extends to the coherence length of the conditional three-photon temporal correlation. Importantly, these dynamic features were not discernible in prior demonstrations. From an alternative perspective, this observation also substantiates the adaptability and adjustability inherent in the generated three-photon state—a crucial attribute for its diverse range of applications.

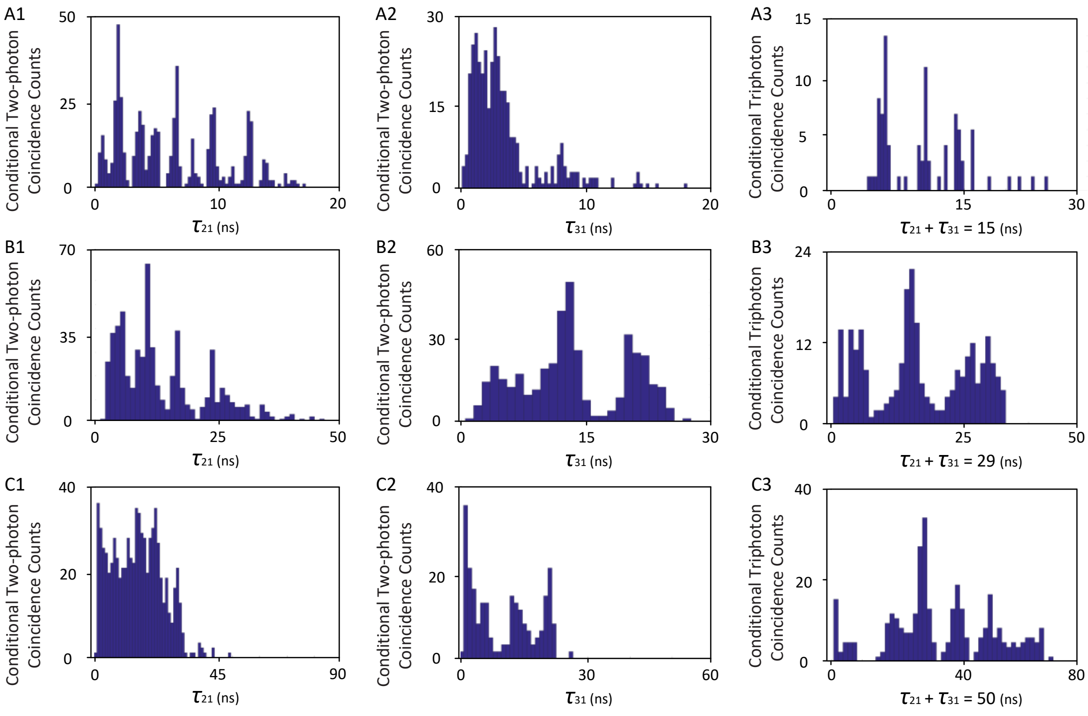

Figure S9: **Conditional two~photon and triphoton coincidence counts, R_2 and R_3, with background accidental subtraction.** Presented here are conditioned two~photon coincidence counts (R_2) and conditional three-photon coincidence counts (R_3) for the scenarios depicted in **Figs. 2A**, **3A**, and **3D** from the main text. Specifically, for **Fig. 2A** in the main text: (**A1**) $R_2(\tau_{21})$ with $\tau_{31} = 2.6$ ns for R_3; (**A2**) $R_2(\tau_{31})$ with $\tau_{21} = 2.0$ ns for R_3; (**A3**) $R_3(\tau_{21} + \tau_{31} = 15.0 \text{ ns})$. For **Fig. 3A** in the main text: (**B1**) $R_2(\tau_{21})$ with $\tau_{31} = 13.0$ ns for R_3; (**B2**) $R_2(\tau_{31})$ with $\tau_{21} = 4.0$ ns for R_3; (**B3**) $R_3(\tau_{21} + \tau_{31} = 29.0 \text{ ns})$ for R_3. For **Fig. 3D** in the main text: (**C1**) $R_2(\tau_{21})$ with $\tau_{31} = 21.0$ ns for R_3; (**C2**) $R_2(\tau_{31})$ with $\tau_{21} = 31.0$ ns for R_3; (**C3**) $R_3(\tau_{21} + \tau_{31} = 50.0 \text{ ns})$ for R_3.

As a W state, the outcome of tracing out the E_{S1}-photons raises an intriguing question. Figures S10A-C respectively report the resulting conditioned two~photon coincidence counts between the remaining E_{S2} and E_{S3} photons for the cases shown in Figs. 2A, 3A, and 3D of the main text. Upon observation, we find that these profiles starkly differ from those illustrated in Figs. 2C, 2D, 3B, and 2F of the main text, as well as Figs. S7C, S8B, S8E, S8F, S9A1-C1, SBA2-C2, S12B, S12C, S13B, and S13C within the SI. The profiles manifested in Fig. S10 below are indeed anticipated, as the E_{S1}-photons do not experience the slow-light effect. As a result, the residual temporal correlations between the remaining E_{S2} and E_{S3} photons assume a nearly symmetrical distribution around the origin of time ($\tau_{32} = 0$).

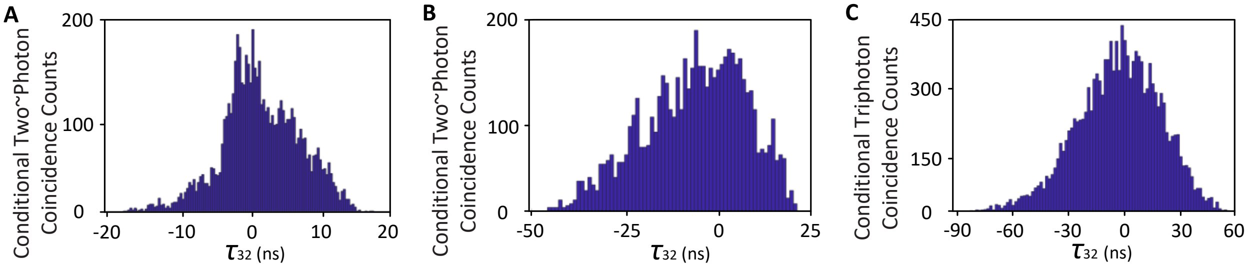

Figure S10: **Conditional two~photon coincidence counts by tracing away E_{S1}-photons.** Derived from the data sets illustrated in **Figs. 2A**, **3A**, and **3D** of the main text, these plots (**A, B & C**) depict conditional two~photon coincidence counts resulting from the removal of E_{S1}-photons. They highlight the partial entanglement or residual quantum correlations between the remaining photons, E_{S2} and E_{S3}.

Procedure for Reconstructing Triphoton Coincidence Counts

Unlike standard two-photon correlation measurements, it's worth noting that a commercially available generic three-photon coincidence circuit is absent in the current market landscape. Consequently, research groups are tasked with constructing their own dedicated three-photon coincidence circuits. As depicted in Fig. S11, we establish a detection system based on two-photon coincidence circuits. Specifically, within a predetermined three-photon correlation time window, we reconstruct three individual single-photon trigger events from $SPCM_1$, $SPCM_2$, and $SPCM_3$. This reconstruction is achieved through the simultaneous detection of two pairs of two-photon coincidence counts, namely $\{E_{S1}, E_{S2}\}$ and $\{E_{S1}, E_{S3}\}$, facilitated by an additional diagnostic $SPCM_D$. Note that in our coincidence counting detection system, the diagnostic photon is used not for post-selection, but rather to exclude accidentals from dual pairs, uncorrelated singles, and dark counts of single-photon detectors.

In practical experimentation, for each recorded three-photon coincidence count, the E_{S1}-photon click serves as a shared start trigger, initiating two electronic pulses I1 from $SPCM_1$. One of these pulses is subjected to a 150-ns delay, as depicted in Fig. S11A. Concurrently, the detections of the E_{S2}-photon and E_{S3}-photon serve as the stop trigger. Here, the electronic pulse I_3 from $SPCM_3$ is delayed by 150 ns relative to the electronic pulse I_2 from $SPCM_2$. With these intricate setups, the

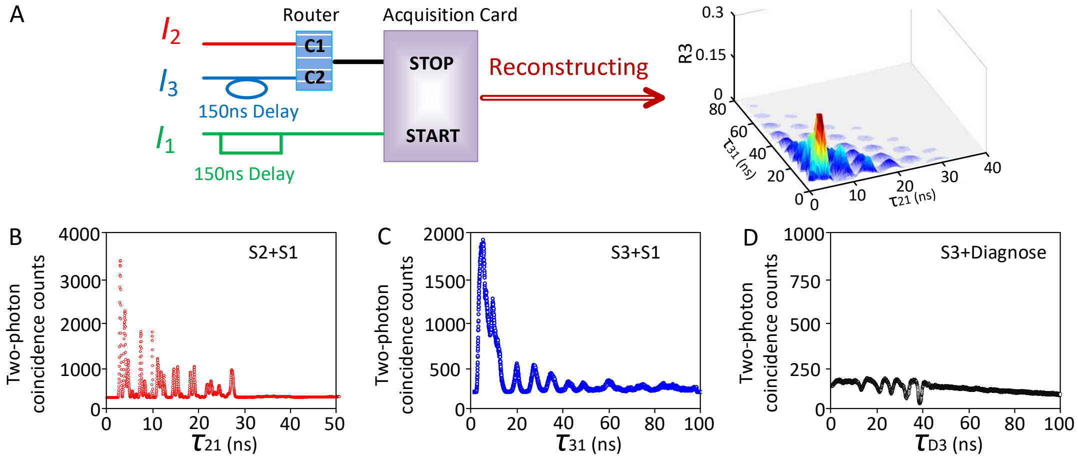

Figure S11: **Home-made three-photon detection system and coincidence counting reconstruction.** (**A**) Illustrated schematic of our home-made detection system that facilitates the reconstruction of three-photon coincidence counting. As an illustrative example, panels (**B–D**) exhibit the recorded two-photon coincidence counts in one experiment, respectively, by $SPCM_1$ and $SPCM_2$, $SPCM_1$ and $SPCM_3$, and $SPCM_3$ and $SPCM_D$. These trigger events are plotted against the relative time differences (τ_2, τ_3 and τ_d) between clicks of the two respetive single-photon detectors. The experimental data was accumulated over 5-minute period, utilizing a time bin width of 0.25 ns for each SPDCM. Additional parameters are set as follows: $P_1 = 4$ mW, $P_2 = 40$ mW, $P_3 = 15$ mW, $\Delta_1 = -2$GHz, $\Delta_2 = -150$MHz, $\Delta_3 = 50$MHz, $\Omega_1 = 300$ MHz, $\Omega_2 = 870$ MHz, and $\Omega_3 = 533$ MHz.

measurement of E_{S1} and E_{S2} photons is conducted first as a function of τ_{21}, followed by the recording of E_{S1} and E_{S3} photons after a 150 ns interval, captured as a function of τ_{31}. This methodology allows for the capture of three-photon temporal correlations within the context of coincidence counting measurements.

To illustrate the functioning of each two-photon coincidence counting component, Fig. S11B-D showcases a representative set of experimental data collected over a span of 5 minutes, employing a time bin width of 0.25 ns for each SPCM. It is evident that the joint detection of E_{S1} and E_{S2} photons elicits a two-photon temporal correlation, varying according to the relative time difference τ_{21} between the clicks of the involved single-photon detectors, $SPCM_1$ and $SPCM_2$ (Fig. S11B). Similarly, the joint detection of E_{S1} and E_{S3} photons unveils a residual temporal correlation, contingent upon the relative triggering time difference τ_{31} between the clicks of the engaged single-photon detectors, $SPCM_1$ and $SPCM_3$ (Fig. S11C). As the diagnostic single-photon detector $SPCM_D$ is triggered by artificial electronic signals, coincident counting between E_{S3} photons and these artificial diagnose signals yields no exact temporal correlation, as demonstrated in Fig. S11D.

Experimentally, capturing authentic triphotons through detection hinges critically on optimizing the phase-matching conditions of the SSWM process. This optimization is achieved by controlling the wavelengths and injection angles of the three input optical driving beams, alongside the directions of triphoton collection. Beyond these arrangements, an additional layer of assurance is established to confirm that the detected triphotons originate exclusively from the intended SSWM process.

This assurance is accomplished by implementing coincident counting detection. Here, the E_{S3} photons are jointly measured with artificially introduced diagnostic signals originating from $SPCM_D$. This joint measurement transpires concurrently with the combined detection of E_{S1} and E_{S2} photons. Utilizing the same reconstruction method outlined earlier, we obtain merely a few

accidental coincidences per minute when employing the two-photon coincidences $\{E_{S1}, E_{S2}\}$ and $\{E_{S3}, E_D\}$ to construct the three-photon histogram. This outcome underscores the absence of any authentic quantum correlation within any two pairs of unrelated two-photon coincidences.

<u>Supplementary Experimental Data</u>

In the experimental domain, we conducted a series of three-photon coincidence counting measurements while varying system parameters. In addition to the data depicted in Figs. 2 and 3 in the main text, we present an additional set of measured data. Illustrated in Fig. S12, we accumulated three-photon coincidence trigger events over the course of 1 hour, utilizing a time bin width of 2.0 ns for each SPCM. Most experimental parameters remain consistent with those detailed in Fig. 2A of the main text, except for P_2 (7 mW), P_3 (7 mW), and OD (45.7).

From the recorded data, it emerges that the triphoton production rate is 100 ± 11 per minute, accompanied by background accidentals of 8 ± 3.1 per minute. Notably, even in this scenario, the triphoton temporal correlation remains within the group-delay regime. This is substantiated by evaluating the conditional two~photon correlations, achieved by tracing away one photon from each triphoton. Fig. S12B and S12C present these conditional two~photon coincidence counts. It is evident that the previously observed Rabi oscillations almost diminish in these two figures.

Illustrated within Fig. S13, we present an additional series of measurements within the group-delay region. A direct comparison with Fig. S12 reveals a significant reduction in the amplitude of the small oscillations observed in the preceding figures.

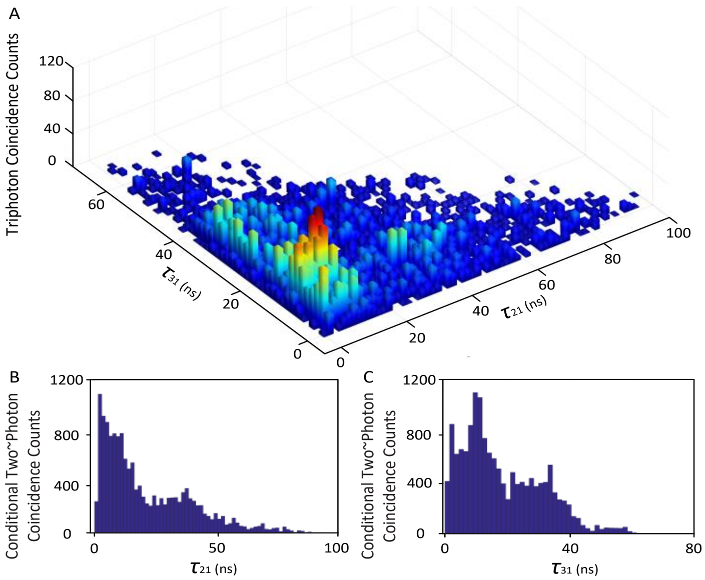

Figure S12: **Triphoton temporal correlation measured in the group-delayed region.** (**A**) Depiction of the histogram representing three-photon coincidence counts spanning 1 hour, utilizing a time-bin width of 2.0 ns for each single-photon detector. The triphoton generation rate amounts to 100 ± 11 per minute, accompanied by background accidental coincidences measured at 8 ± 3.1 per minute. (**B & C**) Conditional two~photon coincidence counts attained by tracing away the E_{S3} or E_{S2} photons from each respective three-photon joint trigger event displayed in panel (**A**). The experimental parameters match those of **Fig. 2A** in the main text, with exceptions being $P_2 = 7$ mW, $P_3 = 7$ mW, $\Omega_2 = 364$ MHz, $\Omega_3 = 364$ MHz, and $OD = 45.7$.

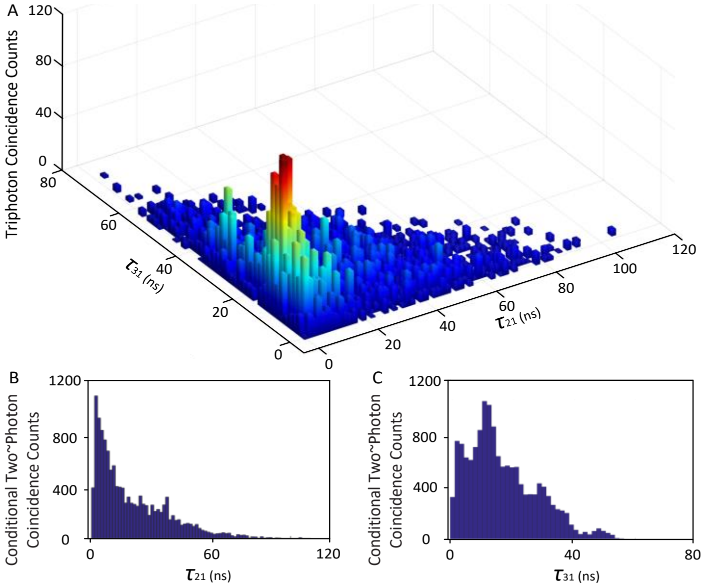

Figure S13: **Triphoton temporal correlation measured in the group-delayed region.** (**A**) Depiction of the histogram representing three-photon coincidence counts spanning 1.5 hours, employing a time-bin width of 1.5 ns for each individual single-photon detector. The triphoton generation rate is determined as 140 ± 15 per minute, with accompanying background accidental coincidences measured at 13 ± 3.4 per minute. (**B & C**) Conditional two~photon coincidence counts achieved through the elimination of the E_{S3} or E_{S2} photons from each respective three-photon joint trigger event presented in panel (**A**). The experimental parameters align with those of **Fig. 2A** in the main text, with alterations such as $P_2 = 6$ mW, $P_3 = 6$ mW, $\Omega_2 = 351$ MHz, $\Omega_3 = 351$ MHz, and $OD = 45.7$.

III. Summary of Diverse Mechanisms for Multiphoton Generation

In this section, we have consolidated the primary experimental demonstrations showcasing the generation of entangled three-photon and multiphoton states, which have been documented up to this point. We've compiled their essential parameters and resulting optical properties in Table S1, providing a convenient point of reference. It is important to acknowledge that our intention is not to list every single experimental report within this compilation. Nonetheless, the reports included here serve as somewhat representative examples of the broader landscape.

IV. Extended Discussion on the Reported Triphoton Source

It is illuminating to investigate the feasibility of the reported triphoton source in generating GHZ-type triphotons entangled in time-energy (and other degrees of freedom) (*56, 57*). To our current understanding, the literature lacks any single proposal for the direct creation of continuous-mode time-energy-entangled GHZ triphotons (*37*). This absence stems from the requirement that, in order to establish such a three-photon GHZ state, two of the photons must be degenerate in all degrees of freedom (*54, 56, 57*).

With regard to our proprietary triphoton source, you might be intrigued by the possible outcome achieved through the arrangement of two of these photons into a degenerate state. Could such an arrangement potentially yield a GHZ state (*37, 56*)? In theory, such a scenario is indeed plausible. However, from a practical perspective, the execution of an experiment of this nature would present substantial challenges. Moreover, considering an alternative perspective, the development of a scheme for the direct generation of continuous-mode triphoton and multi-photon states entangled in time-energy and space-momentum domains still necessitates additional in-depth research efforts.

Table S1 – Experiments on Multiphoton Entanglement Generation

Class	State	Counts per hour	Reference
Triphoton Source	time-energy (cascaded SPDCs)	6.2	*(10)*
	time-energy (cascaded SPDCs)	7	*(11)*
	polarization GHZ (cascaded SPDCs)	744	*(12)*
	**W for various degrees of freedom (SSWM)**	**7500**	***This work***
Specific Multiphoton State Generation	3-photon polarization GHZ (SPDC)	24	*(4)*
	4-photon polarization GHZ (SPDC)	69	*(5)*
	4-photon polarization GHZ (SPDC)	300	*(19)*
	4-photon polarization (SPDC)	175	*(21)*
	5-photon polarization GHZ (SPDC)	10	*(16)*
	3-photon polarization W (SPDC)	5220	*(17)*
	4-photon polarization Dicke (SPDC)	3600	*(7)*
	3-photon discrete-energy W (SFWM)	75	*(57)*
	4-photon polarization GHZ (SFWM)	2088	*(22)*
	4-photon polarization GHZ (SFWM)	6084	*(23)*

Table S1： **Comparison of notable experiments on entangled multiphoton generation using indirect methods, as outlined in the introduction section of the main text, with our direct generation based on SSWM.** Even without any optimization, our experiment has already set a record in generation rate.

REFERENCES AND NOTES

1. J.-W. Pan, Z.-B. Chen, C.-Y. Lu, H. Weinfurter, A. Zeilinger, M. Zukowski, Multiphoton entanglement and interferometry. *Rev. Mod. Phys.* **84**, 777–838 (2012).

2. N. Friis, G. Vitagliano, M. Malik, M. Huber, Entanglement certification from theory to experiment. *Nat. Rev. Phys.* **1**, 72–87 (2019).

3. M. Erhard, M. Krenn, A. Zeilinger, Advances in high-dimensional quantum entanglement. *Nat. Rev. Phys.* **2**, 365–381 (2020).

4. D. Bouwmeester, J.-W. Pan, M. Daniell, H. Weinfurter, A. Zeilinger, Observation of three-photon Greenberger-Horne-Zeilinger entanglement. *Phys. Rev. Lett.* **82**, 1345–1349 (1999).

5. J.-W. Pan, D. Bouwmeester, S. Gasparoni, G. Weihs, A. Zeilinger, Experimental demonstration of four-photon entanglement and high-fidelity teleportation. *Phys. Rev. Lett.* **86**, 4435–4438 (2001).

6. M. Eibl, N. Kiesel, M. Bourennane, C. Kurtsiefer, H. Weinfurther, Experimental realization of a three-qubit entangled W state. *Phys. Rev. Lett.* **92**, 077901 (2004).

7. N. Kiesel, C. Schmid, G. Toth, E. Solano, H. Weinfurther, Experimental observation of four-photon entangled Dicke state with high fidelity. *Phys. Rev. Lett.* **98**, 063604 (2007).

8. C. Reimer, M. Kues, P. Roztocki, B. Wetzel, F. Grazioso, B. E. Little, S. T. Chu, T. Johnson, Y. Bromberg, L. Caspani, D. J. Moss, R. Morandotti, Generation of multiphoton entangled quantum states by means of integrated frequency combs. *Science* **351**, 1176–1180 (2016).

9. J. Wen, E. Oh, S. Du, Tripartite entanglement generation via four-wave mixings: Narrowband triphoton W state. *J. Opt. Soc. Am. B* **27**, A11–A20 (2010).

10. H. Hubel, D. R. Hamel, A. Fedrizzi, S. Ramelow, K. J. Resch, T. Jennewein, Direct generation of photon triplets using cascaded photon-pair sources. *Nature* **466**, 601–603 (2010).

11. L. K. Shalm, D. R. Hamel, Z. Yan, C. Simon, K. J. Resch, T. Jennewein, Three-photon energy-time entanglement. *Nat. Phys.* **9**, 19–22 (2013).

12. D. R. Hamel, L. K. Shalm, H. Hübel, A. J. Miller, F. Marsili, V. B. Verma, R. P. Mirin, S. W. Nam, K. J. Resch, T. Jennewein. Direct generation of three-photon polarization entanglement. *Nat. Photonics* **8**, 801–807 (2014).

13. T. E. Keller, M. H. Rubin, Y. Shih, L.-A. Wu, Theory of the three-photon entangled state. *Phys. Rev. A* **57**, 2076–2079 (1998).

14. J. Wen, P. Xu, M. H. Rubin, Y. Shih, Transverse correlations in triphoton entanglement: Geometrical and physical optics. *Phys. Rev. A* **76**, 023828 (2007).

15. J. Rarity, P. Tapster, Three-particle entanglement from entangled photon pairs and a weak coherent state. *Phys. Rev. A* **59**, R35–R38 (1999).

16. Z. Zhao, Y.-A. Chen, A.-N. Zhang, T. Yang, H. J. Briegel, J.-W. Pan, Experimental demonstration of five-photon entanglement and open-destination teleportation. *Nature* **430**, 54–58 (2004).

17. H. Mikami, Y. Li, K. Fukuoka, T. Kobayashi, New high-efficiency source of a three-photon W state and its full characterization using quantum state tomography. *Phys. Rev. Lett.* **95**, 150404 (2005).

18. C. K. Hong, Z. Y. Ou, L. Mandel, Measurement of subpicosecond time intervals between two photons by interference. *Phys. Rev. Lett.* **59**, 2044–2046 (1987).

19. M. Eibl, S. Gaertner, M. Bourennane, C. Kurtsiefer, M. Zukowski, H. Weinfurther, Experimental observation of four-photon entanglement from parametric down-conversion. *Phys. Rev. Lett.* **90**, 200403 (2003).

20. H. de Riedmatten, V. Scarani, I. Marcikic, A. Acin, W. Tittel, H. Zbinden, N. Gisin, Two independent photon pairs versus four-photon entangled states in parametric down conversion. *J. Mod. Opt.* **51**, 1637–1649 (2003).

21. M. Bourennane, M. Eibl, S. Gaertner, C. Kurtsiefer, A. Cabello, H. Weinfurther, Decoherence-free quantum information processing with four-photon entangled states. *Phys. Rev. Lett.* **92**, 107901 (2004).

22. J. Park, H. Kim, H. S. Moon, Four-photon Greenberger-Horne-Zeilinger entanglement via collective two-photon coherence in Doppler-broadened atoms. *Adv. Quantum Technol.* **4**, 2000152 (2021).

23. J. Park, H. S. Moon, Generation of a bright four-photon entangled state from a warm atomic ensemble via inherent polarization entanglement. *Appl. Phys. Lett.* **120**, 024001 (2022).

24. M. Corna, K. Garay-Palmett, A. B. U'Ren, Experimental proposal for the generation of entangled photon triplets by third-order spontaneous parametric downconversion in optical fibers. *Opt. Lett.* **36**, 190–192 (2011).

25. N. A. Borshchevskaya, K. G. Katamadze, S. P. Kulik, M. V. Fedorov, Three-photon generation by means of third-order spontaneous parametric down-conversion in bulk crystals. *Laser Phys. Lett.* **12**, 115404 (2015).

26. C. W. S. Chang, C. Sabín, P. Forn-Díaz, F. Quijandría, A. M. Vadiraj, I. Nsanzineza, G. Johansson, C. M. Wilson, Observation of three-photon spontaneous parametric down-conversion in a superconducting parametric cavity. *Phys. Rev. X* **10**, 011011 (2020).

27. M. Fleischhauer, A. Imamoglu, J. P. Marangos, Electromagnetically induced transparency: Optics in coherent media. *Rev. Mod. Phys.* **77**, 733–673 (2005).

28. S. Du, J. Wen, M. H. Rubin, Narrowband biphoton generation near atomic resonance. *J. Opt. Soc. Am. B* **25**, C98–C108 (2008).

29. V. Balic, D. A. Braje, P. Kolchin, G. Y. Yin, S. E. Harris, Generation of paired photons with controllable waveforms. *Phys. Rev. Lett.* **94**, 183601 (2005).

30. S. Du, P. Kolchin, C. Belthangady, G. Y. Yin, S. E. Harris, Subnatural linewidth biphotons with controllable temporal length. *Phys. Rev. Lett.* **100**, 183603 (2008).

31. C. Shu, P. Chen, T. K. A. Chow, L. Zhu, Y. Xiao, M. M. T. Loy, S. Du, Subnatural-linewidth biphotons from a Doppler-broadened hot atomic vapour cell. *Nat. Commun.* **7**, 12783 (2016).

32. J. Wen, S. Du, M. H. Rubin, Biphoton generation in a two-level atomic ensemble. *Phys. Rev. A* **75**, 033809 (2007).

33. J. Wen, S. Du, M. H. Rubin, Spontaneous parametric down-conversion in a three-level system. *Phys. Rev. A* **76**, 013825 (2007).

34. J. Wen, S. Du, Y. Zhang, M. Xiao, M. H. Rubin, Nonclassical light generation via a four-level inverted-Y system. *Phys. Rev. A* **77**, 033816 (2008).

35. H. Kang, G. Hernandez, Y. Zhu, Slow-light six-wave mixing at low light intensities. *Phys. Rev. Lett.* **93**, 073601 (2004).

36. Y. Zhang, A. W. Brown, M. Xiao, Opening four-wave mixing and six-wave mixing channels via dual electromagnetically induced transparency windows. *Phys. Rev. Lett.* **99**, 123603 (2007).

37. J. Wen, M. H. Rubin, Distinction of tripartite Greenberger-Horne-Zeilinger and *W* states entangled in time (or energy) and space. *Phys. Rev. A* **79**, 025802 (2009).

38. M. D. Reid, D. F. Walls, Violations of classical inequalities in quantum optics. *Phys. Rev. A* **34**, 1260–1276 (1986).

39. A. V. Belinskii, D. N. Klyshko, Interference of light and Bell's theorem. *Phys. Usp.* **36**, 653–693 (1993).

40. D. Zhang, Y. Cai, Z. Zheng, D. Barral, Y. Zhang, M. Xiao, K. Bencheikh, Non-Gaussian nature and entanglement of spontaneous parametric nondegenerate triple-photon generation. *Phys. Rev. A* **103**, 013704 (2021).

41. K. Li, Y. Cai, J. Wu, Y. Liu, S. Xiong, Y. Li, Y. Zhang, Three-body topology entanglement generation via a six-wave mixing: Competing and coexisting of linear and nonlinear Optics responses in triphoton temporal correlation. *Adv. Quantum Technol.* **3**, 1900119 (2020).

42. S. Zhang, W. Li, K. Li, Y. Li, F. Mu, Y. Feng, Y. Liu, Y. Zhang, Triphoton correlations in six-wave mixing. *Ann. Phys.* **412**, 168000 (2020).

43. Z. Nie, H. Zheng, P. Li, Y. Yang, Y. Zhang, M. Xiao, Interacting multiwave mixing in a five-level atomic system. *Phys. Rev. A* **77**, 063829 (2008).

44. H. X. Chen, M. Z. Qin, Y. Q. Zhang, X. Zhang, F. Wen, J. M. Wen, Y. P. Zhang, Parametric amplification of dressed multi-wave mixing in an atomic ensemble. *Laser. Phys. Lett.* **11**, 045201 (2014).

45. K. Li, D. Zhang, F. Raza, Z. Zhang, P. Puttapirat, Y. Liu, Y. Zhang, Multi-contact switch using double-dressing regularity of probe, fluorescence, and six-wave mixing in a Rydberg atom. *J. Chem. Phys.* **149**, 074310 (2018).

46. S. Yun, J. Wen, P. Xu, M. Xiao, S. N. Zhu, Generation of frequency-correlated narrowband biphotons from four-wave mixing in cold atoms. *Phys. Rev. A* **82**, 063830 (2010).

47. J. Wen, Y. H. Zhai, S. Du, M. Xiao, Engineering biphoton wave packets with an electromagnetically induced grating. *Phys. Rev. A* **82**, 043814 (2010).

48. S. Du, E. Oh, J. Wen, M. H. Rubin, Four-wave mixing in three-level systems: Interference and entanglement. *Phys. Rev. A* **76**, 013803 (2007).

49. S. Du, J. Wen, M. H. Rubin, G. Y. Yin, Four-wave mixing and biphoton generation in a two-level system. *Phys. Rev. Lett.* **98**, 53601 (2007).

50. J. Wen, M. H. Rubin, Transverse effects in paired-photon generation via an electromagnetically induced transparency medium. I. Perturbation theory. *Phys. Rev. A* **74**, 023808 (2006).

51. M. H. Rubin, D. N. Klyshko, Y.-H. Shih, A. V. Sergienko, Theory of two-photon entanglement in type-II optical parametric down-conversion. *Phys. Rev. A* **50**, 5122–5133 (1994).

52. D. N. Klyshko, *Photons and Nonlinear Optics* (Gordon and Breach, 1988).

53. J. Wen, M. H. Rubin, Y. Shih, Transverse correlations in multiphoton entanglement. *Phys. Rev. A* **76**, 45802 (2007).

54. J. Wen, S. Du, M. Xiao, Improving spatial resolution in quantum imaging beyond the Rayleigh diffraction limit using multiphoton W entangled states. *Phys. Lett. A* **374**, 3908–3911 (2010).

55. S. L. Braunstein, P. van Loock, Quantum information with continuous variables. *Rev. Mod. Phys.* **77**, 513–577 (2005).

56. J. Wen, M. H. Rubin, Y. H. Shih, Spatial resolution enhancement in quantum imaging beyond the diffraction limit using entangled photon-number state. arXiv:0812.2032 (2008).

57. B. Fang, M. Menotti, M. Liscidini, J. E. Sipe, V. O. Lorenz, Three-photon discrete-energy-entangled W state in an optical fiber. *Phys. Rev. Lett.* **123**, 070508 (2019).
